# Data on medicinal plants in the records of Latvian folk medicine from the 19th century

**DOI:** 10.1016/j.dib.2019.105024

**Published:** 2019-12-20

**Authors:** Inga Sile, Edita Romane, Sanita Reinsone, Baiba Maurina, Dace Tirzite, Maija Dambrova

**Affiliations:** a Department of Dosage Form Technology, Riga Stradins University, 16 Dzirciema Str., Riga, LV-1007, Latvia; b Institute of Literature, Folklore and Art of the University of Latvia, 3 Mūkusalas Str., Riga, LV-1423, Latvia; c Latvian Institute of Organic Synthesis, 21 Aizkraukles Str., LV-1006, Riga, Latvia; d Department of Pharmaceutical Chemistry, Riga Stradins University, 16 Dzirciema Str., Riga, LV-1007, Latvia

**Keywords:** Medicinal plants, Folk medicine, Ethnobotany, Records of Latvian folk medicine

## Abstract

The data presented in this article is in support of the research paper “Medicinal plants and their uses recorded in the Archives of Latvian Folklore from the 19th century” [1]. This article provides the list of plant species and disorders treated with medicinal plants mentioned in the records of Latvian folk medicine and used by indigenous people of Latvia in the 19th century. In total, the data include 211 genera belonging to 71 plant families. The accepted scientific names of plant species, plant parts used, dosage forms of herbal medicines, and routes of administration are reported in the table. Plant uses are grouped into one of the 17 categories based on the body systems and psychological and social problems. The frequency of citations is indicated for each use of medicinal plant.

**Table:** Specifications Table

Subject	Biology
Specific subject area	Medicinal plants and their uses
Type of data	Table
How data were acquired	The records of Latvian folk medicine were collected in Excel file and systematically reviewed
Data format	Raw and analysed
Parameters for data collection	More than 40000 records from the Archives of Latvian Folklore were analysed to select those containing information about historical evidence of medicinal plant usage in the territory of Latvia during the 19th century. Over 1900 records were found in the folklore materials.
Description of data collection	Each plant was identified by scientific name according to The Plant List website. Following The International Classification of Primary Care, plant uses were grouped into one of 17 medicinal use categories. Additionally, the plant parts used, dosage forms of herbal medicines and routes of administration were analysed. Based on current available scientific information, medicinal plants for which herbal monographs have been developed are indicated in the table.
Data source location	Riga, Latvia
Data accessibility	With the article
Related research article	I. Sile, E. Romane, S. Reinsone, B. Maurina, D. Tirzite, M. Dambrova, 2020. Medicinal plants and their uses recorded in the Archives of Latvian Folklore from the 19th century. J. Ethnopharmacol. 249, 112378. https://doi.org/10.1016/j.jep.2019.112378.

**Table:** 

**Value of the Data** • The analysis provides a list of medicinal plants, including their health benefits and applications, used by Latvia's indigenous people. • The data can be used as a reference to indications, plant parts, preparations, and routes of administration mentioned of the specific plant species that were utilized for medicinal purposes. • These data can be useful in comparative ethnobotanical studies about medicinal plants and their uses, including cross-cultural comparisons. • Plant species and their uses described in the records of Latvian folk medicine could be potentially useful for future herbal medicine research.

## 1 Data description

The data were analysed for a recent study [1], based upon information collected from the records of Latvian folk medicine available in the Archives of Latvian Folklore. This is the first comprehensive overview of the Latvian folk herbal traditions in the 19th century. The comprehensive data of plant species collected in the records of Latvian folk medicine are presented in Table 1. The table reports the Latvian names of plants mentioned in the records, currently accepted names in Latin, therapeutic uses of recorded plant species, as well as the plant parts used, dosage forms of herbal medicines, and routes of administration. Medicinal plants having herbal monograph published by the European Medicines Agency and those included in the book Herbal Drugs and Phytopharmaceuticals [2] are highlighted.

## 2 Experimental design, materials, and methods

Data on plants and their uses were collected from the records of Latvian folk medicine, the Archives of Latvian Folklore (ALF). In total, more than 40000 records were reviewed to select those containing information about plant usage in human medicine. A part of the records was obtained from the collections digitized by the Digital Archives of Latvian Folklore [3]. Some records were collected from the four published volumes titled “Latvian Folk Beliefs” compiled by the folklorist P. Šmits [4]. This compilation contains 36790 records and it is also digitized and published [5].

For each plant identified in the records, scientific family, genus, and species names are shown in the table. Plant names are updated to the currently accepted names in Latin following The Plant List website [6]. Family names are arranged using the Angiosperm Phylogeny Group 4 classification [7]. Local plant names in official Latvian language are provided according to the Encyclopedia Nature of Latvia [8].

The International Classification of Primary Care (ICPC; [9]) accepted by the World Health Organization was used for disease classification. Following the ICPC, plant uses were grouped into one of the 17 categories based on the systems of the human body, psychological or social problems, as follows: 1) General and unspecified; 2) Blood, blood-forming organs, lymphatics and spleen; 3) Digestive; 4) Eye; 5) Ear; 6) Circulatory; 7) Musculoskeletal; 8) Neurological; 9) Psychological; 10) Respiratory; 11) Skin; 12) Endocrine, metabolic and nutritional; 13) Urology; 14) Pregnancy, childbirth and family planning; 15) Female genital system and breast; 16) Male genital system; and 17) Unspecified medicinal disorders, the last of which was used when it was not possible to code the disease. The number of citations was indicated for each use of medicinal plant.

Plant parts, preparations of the medicine, and routes of administration were described for each plant mentioned in the records of folk medicine. When there was no mention of plant parts, preparations or administration, the category “unspecified” was used.

Medicinal plants from the records of Latvian folk medicine which are included in the European Union herbal monographs published by the European Medicines Agency [10] or in the monographs of Herbal Drugs and Phytopharmaceuticals [2] are indicated in the table.
