# Supplementary material for: Data on medicinal plants in the records of Latvian folk medicine from the 19th century
Source: Data Brief. 2019 Dec 20;28:105024. doi: 10.1016/j.dib.2019.105024 (PMC6940611; doi:10.1016/j.dib.2019.105024)
Supplement: Multimedia component 1 [file mmc1.docx]

Table 1

List of plants identified in the records of Latvian folk medicine to either species or genus level.

Note: Ext. – external use; O. ad. – oral administration; Unsp – unspecified.

* Assessment by the European Medicines Agency’s Committee on Herbal Medicinal Products. Status type - C: Ongoing call for scientific data; D: Draft under discussion; F: Assessment finalised; P: Draft published.

** Herbal drugs listed in: M. Wichtl, Herbal Drugs and Phytopharmaceuticals: A Handbook for Practice on a Scientific Basis, Third ed., CRC press, Stuttgart, 2004.

# The use of plant not identified in the records of Latvian folk medicine.

| Family | Scientific plant name | Local plant name | Part used | Herbal preparation | Administration | Disorder | Therapeutic uses | No. of citations | Assessment by the EMA* | Wichtl (2004)** |
| --- | --- | --- | --- | --- | --- | --- | --- | --- | --- | --- |
| Acoraceae | *Acorus calamus* L. | *smaržīgā kalme* | Leaves, rhizome | Bath, decoction, decoction with honey, raw material, tea, tincture | Ext., O. ad. | Digestive | Diarrhoea | 3 |  | × (rhizome) |
|  |  |  |  |  |  |  | Dysentery | 3 |  |  |
|  |  |  |  |  |  |  | Generalized abdominal pain | 3 |  |  |
|  |  |  |  |  |  |  | Hernia | 2 |  |  |
|  |  |  |  |  |  |  | Stomach function disorders | 2 |  |  |
|  |  |  |  |  |  |  | Teething problem | 1 |  |  |
|  |  |  |  |  |  |  | Toothache | 3 |  |  |
|  |  |  |  |  |  | Endocrine, metabolic and nutritional | Loss of appetite | 2 |  |  |
|  |  |  |  |  |  | General and unspecified | Fever | 1 |  |  |
|  |  |  |  |  |  | Musculoskeletal | Bone pain | 2 |  |  |
|  |  |  |  |  |  |  | Rheumatism | 6 |  |  |
|  |  |  |  |  |  | Neurological | Headache | 1 |  |  |
|  |  |  |  |  |  | Psychological | Feeling anxious/ nervous/ tense | 1 |  |  |
|  |  |  |  |  |  |  | Tobacco abuse | 1 |  |  |
|  |  |  |  |  |  | Respiratory | Chest pain | 1 |  |  |
|  |  |  |  |  |  |  | Respiratory complaint | 2 |  |  |
|  |  |  |  |  |  | Skin | Hair loss | 1 |  |  |
| Amaranthaceae | *Atriplex* L. | *balodene* | Aerial parts | Raw material | Ext. | Skin | Athlete's foot | 1 |  |  |
|  | *Beta vulgaris* L. | *parastā biete* | Leaves, roots | Juice, raw material | Ext., O. ad. | Digestive | Stomach function disorders | 1 |  |  |
|  |  |  |  |  |  | General and unspecified | Fever | 1 |  |  |
|  |  |  |  |  |  |  | Tuberculosis | 2 |  |  |
|  |  |  |  |  |  | Neurological | Epilepsy | 1 |  |  |
|  |  |  |  |  |  |  | Headache | 1 |  |  |
|  |  |  |  |  |  | Skin | Abscess | 1 |  |  |
|  |  |  |  |  |  |  | Cuts and wounds | 2 |  |  |
| Amaryllidaceae | *Allium cepa* L. | *dārza sīpols* | Bulb | Baked, bath, decoction, juice, raw material, steamed material, tea, tincture, with vinegar | Ext., O. ad. | Blood, blood forming organs, lymphatics, spleen | Spleen problems | 1 |  |  |
|  |  |  |  |  |  | Circulatory | Cardiovascular problems | 1 |  |  |
|  |  |  |  |  |  | Digestive | Dysentery | 1 |  |  |
|  |  |  |  |  |  |  | Toothache | 7 |  |  |
|  |  |  |  |  |  |  | Worms | 1 |  |  |
|  |  |  |  |  |  | Ear | Earache | 6 |  |  |
|  |  |  |  |  |  | Eye | Eye problems | 1 |  |  |
|  |  |  |  |  |  | General and unspecified | Bleeding | 1 |  |  |
|  |  |  |  |  |  |  | Fever | 1 |  |  |
|  |  |  |  |  |  |  | Bacterial infection | 1 |  |  |
|  |  |  |  |  |  | Respiratory | Cough | 5 |  |  |
|  |  |  |  |  |  |  | Diphtheria | 1 |  |  |
|  |  |  |  |  |  |  | Runny nose | 2 |  |  |
|  |  |  |  |  |  |  | Sore throat | 2 |  |  |
|  |  |  |  |  |  |  | Voice loss | 1 |  |  |
|  |  |  |  |  |  | Skin | Snake bite | 5 |  |  |
|  |  |  |  |  |  |  | Abscess | 18 |  |  |
|  |  |  |  |  |  |  | Burns | 2 |  |  |
|  |  |  |  |  |  |  | Ulcer | 1 |  |  |
|  |  |  |  |  |  |  | Hair loss | 2 |  |  |
|  |  |  |  |  |  |  | Insect bite | 2 |  |  |
|  |  |  |  |  |  |  | Lichen | 1 |  |  |
|  |  |  |  |  |  |  | Athlete's foot | 1 |  |  |
|  |  |  |  |  |  |  | Warts | 3 |  |  |
|  |  |  |  |  |  | Urology | Cystitis | 1 |  |  |
|  | *Allium sativum* L. | *ķiploks* | Bulb | Decoction, juice, raw material, with milk | Ext., O. ad. | Digestive | Hernia | 1 | F: EMA/HMPC/7685/2013  Cough and cold  Atherosclerosis^#^ | × (bulb powder) |
|  |  |  |  |  |  |  | Toothache | 4 |  |  |
|  |  |  |  |  |  |  | Worms | 2 |  |  |
|  |  |  |  |  |  | Ear | Earache | 4 |  |  |
|  |  |  |  |  |  | General and unspecified | Bacterial infection | 1 |  |  |
|  |  |  |  |  |  | Musculoskeletal | Cramps | 1 |  |  |
|  |  |  |  |  |  | Respiratory | Laryngo-tracheo-bronchitis | 1 |  |  |
|  |  |  |  |  |  | Skin | Snake bite | 1 |  |  |
|  |  |  |  |  |  |  | Abscess | 5 |  |  |
|  |  |  |  |  |  |  | Ulcer | 1 |  |  |
|  |  |  |  |  |  |  | Splinter | 1 |  |  |
|  |  |  |  |  |  |  | Insect bite | 1 |  |  |
|  |  |  |  |  |  |  | Cuts and wounds | 1 |  |  |
|  |  |  |  |  |  |  | Rash | 1 |  |  |
|  |  |  |  |  |  |  | Lichen | 1 |  |  |
|  |  |  |  |  |  |  | Warts | 1 |  |  |
| Apiaceae | *Angelica sylvestris* L. | *meža zirdzene* | Unsp | Unsp | Unsp | Unsp | Unsp | 1 |  | × (root of *A. Sylvestris*, *A. archangelica*) |
|  | *Apium graveolens* L. | *selerija* | Unsp | Decoction | O. ad. | Musculoskeletal | Rheumatism | 1 |  | × (fruit) |
|  | *Carum carvi* L. | *parastā ķimene* | Fruits | Baked, decoction, tea | Ext., O. ad. | Digestive | Diarrhoea | 1 | F: EMA/HMPC/715092/2013  Bloating and flatulence^#^ | × (fruit) |
|  |  |  |  |  |  |  | Heartburn | 2 |  |  |
|  |  |  |  |  |  | Ear | Earache | 1 |  |  |
|  |  |  |  |  |  | Endocrine, metabolic and nutritional | Loss of appetite | 1 |  |  |
|  |  |  |  |  |  | General and unspecified | Fever | 1 |  |  |
|  |  |  |  |  |  | Respiratory | Respiratory complaint | 2 |  |  |
|  | *Cicuta virosa* L. | *indīgais velnarutks* | Roots | Raw material, tea | Ext., O. ad. | Male genital system | Male sexual function complaint | 1 |  |  |
|  |  |  |  |  |  | Skin | Lichen | 1 |  |  |
|  |  |  |  |  |  |  | Cuts and wounds | 1 |  |  |
|  | *Daucus* L. | *burkāns* | Aerial parts, roots | Decoction, juice, raw material, tea | O. ad. | Digestive | Jaundice | 2 |  |  |
|  |  |  |  |  |  |  | Worms | 1 |  |  |
|  |  |  |  |  |  | General and unspecified | Tuberculosis | 2 |  |  |
|  |  |  |  |  |  | Respiratory | Chest pain | 1 |  |  |
|  | *Levisticum officinale* W.D.J. Koch | *ārstniecības lupstājs* | Flowers, herb, leaves | Bath, compresses, decoction, raw material, tea, with urine | Ext., O. ad. | Blood, blood forming organs, lymphatics, spleen | Blood problems | 1 | F: EMA/HMPC/524621/2011 (root)  Adjuvant in minor urinary complaints^#^ | × (root) |
|  |  |  |  |  |  | Circulatory | Cardiovascular problems | 1 |  |  |
|  |  |  |  |  |  | Digestive | Sharp, throbbing abdominal pain | 1 |  |  |
|  |  |  |  |  |  |  | Toothache | 1 |  |  |
|  |  |  |  |  |  | Female genital system and breast | Excessive menstrual bleeding | 1 |  |  |
|  |  |  |  |  |  | General and unspecified | Swelling | 11 |  |  |
|  |  |  |  |  |  | Musculoskeletal | Bone pain | 1 |  |  |
|  |  |  |  |  |  |  | Rheumatism | 1 |  |  |
|  |  |  |  |  |  | Respiratory | Respiratory complaint | 2 |  |  |
|  |  |  |  |  |  |  | Sore throat | 2 |  |  |
|  |  |  |  |  |  | Skin | Erysipelas | 1 |  |  |
|  |  |  |  |  |  |  | Scabies | 1 |  |  |
|  |  |  |  |  |  |  | Snake bite | 1 |  |  |
|  | *Petroselinum* Hill | *pētersīlis* | Aerial parts, roots | Decoction, tea, with wine | Ext., O. ad. | Digestive | Diarrhoea | 1 |  | × (fruit, root of *P. crispum*) |
|  |  |  |  |  |  | General and unspecified | Swelling | 1 |  |  |
|  |  |  |  |  |  |  | Fever | 1 |  |  |
|  |  |  |  |  |  | Pregnancy, childbirth, family planning | Induced abortion | 1 |  |  |
|  |  |  |  |  |  | Psychological | Learning problems | 1 |  |  |
|  |  |  |  |  |  | Skin | Cuts and wounds | 1 |  |  |
|  |  |  |  |  |  | Urology | Kidney problems | 1 |  |  |
|  |  |  |  |  |  |  | Urinary retention | 1 |  |  |
|  |  |  |  |  |  |  | Urination problems | 2 |  |  |
|  | *Pimpinella anisum* L. | *parastais anīss* | Fruits | Drops | O. ad. | Respiratory | Chest pain | 1 | F: EMA/HMPC/321184/2012  Cough and cold  Bloating and flatulence^#^ | × (fruit) |
|  |  |  |  |  |  |  | Cough | 1 |  |  |
|  |  |  |  |  |  |  | Acute upper respiratory infection | 1 |  |  |
|  | *Pimpinella* L. | *noraga* | Roots | Decoction, tea | O. ad. | Circulatory | Cardiovascular problems | 1 |  | × (root of *P. major*) |
|  |  |  |  |  |  | Digestive | Sharp, throbbing abdominal pain | 1 |  |  |
|  | *Sium latifolium* L. | *platlapu cemere* | Unsp | Unsp | O. ad. | Digestive | Generalized abdominal pain | 1 |  |  |
| Apocynaceae | *Vinca minor* L. | *mazā kapmirte* | Unsp | Unsp | Unsp | Unsp | Unsp | 1 |  |  |
| Asparagaceae | *Albuca bracteata* (Thunb.) J.C.Manning & Goldblatt | *neīstais jūras sīpols* | Bulb, leaves | Decoction, juice, raw material, with cream | Ext., O. ad. | Neurological | Headache | 1 |  |  |
|  |  |  |  |  |  | Respiratory | Cough | 3 |  |  |
|  | *Convallaria majalis* L. | *parastā kreimene* | Flowers | Tincture | O. ad. | Digestive | Sharp, throbbing abdominal pain | 1 |  |  |
|  |  |  |  |  |  | Psychological | Feeling anxious/ nervous/ tense | 1 |  |  |
|  | *Polygonatum odoratum* (Mill.) Druce | *ārstniecības mugurene* | Unsp | Unsp | Unsp | Musculoskeletal | Bone pain | 1 |  |  |
| Asphodelaceae | *Aloe* sp. L. | *alveja* | Leaves | Boiled material, decoction, juice, raw material, tincture, with honey, with milk, with oil | Ext., O. ad. | Circulatory | Heart pain | 1 | F: EMA/HMPC/625788/2015  Constipation | × ( dried juice of the leaves of *A. barbadensis*, *A. capensis*) |
|  |  |  |  |  |  | Digestive | Constipation | 1 |  |  |
|  |  |  |  |  |  |  | Hernia | 1 |  |  |
|  |  |  |  |  |  |  | Sharp, throbbing abdominal pain | 1 |  |  |
|  |  |  |  |  |  |  | Vomiting | 1 |  |  |
|  |  |  |  |  |  | General and unspecified | Tuberculosis | 6 |  |  |
|  |  |  |  |  |  | Respiratory | Chest pain | 11 |  |  |
|  |  |  |  |  |  |  | Cough | 5 |  |  |
|  |  |  |  |  |  |  | Respiratory complaint | 1 |  |  |
|  |  |  |  |  |  |  | Shortness of breath | 1 |  |  |
|  |  |  |  |  |  | Skin | Abscess | 2 |  |  |
|  |  |  |  |  |  |  | Burns | 1 |  |  |
|  |  |  |  |  |  |  | Cuts and wounds | 8 |  |  |
|  |  |  |  |  |  |  | Scar | 1 |  |  |
| Asteraceae | *Achillea millefolium* L. | *parastais pelašķis* | Aerial parts, flowers, roots | Decoction, juice, raw material, tea, tincture | Ext., Inhalation, O. ad. | Digestive | Diarrhoea | 1 | F: EMA/HMPC/143949/2010 (flower)  F: EMA/HMPC/290284/2009 (herb)  Bloating and flatulence  Small superficial wounds  Spasm associated with menstrual periods^#^  Loss of appetite^#^ | × (aerial part) |
|  |  |  |  |  |  |  | Flatulence | 1 |  |  |
|  |  |  |  |  |  |  | Generalized abdominal pain | 4 |  |  |
|  |  |  |  |  |  |  | Hernia | 1 |  |  |
|  |  |  |  |  |  |  | Toothache | 1 |  |  |
|  |  |  |  |  |  | Ear | Earache | 1 |  |  |
|  |  |  |  |  |  | General and unspecified | Bleeding | 21 |  |  |
|  |  |  |  |  |  |  | Tuberculosis | 4 |  |  |
|  |  |  |  |  |  | Musculoskeletal | Cramps | 1 |  |  |
|  |  |  |  |  |  | Psychological | Feeling anxious/ nervous/ tense | 1 |  |  |
|  |  |  |  |  |  | Respiratory | Cough | 35 |  |  |
|  |  |  |  |  |  |  | Runny nose | 1 |  |  |
|  |  |  |  |  |  |  | Sore throat | 1 |  |  |
|  |  |  |  |  |  | Skin | Athlete's foot | 3 |  |  |
|  |  |  |  |  |  |  | Boils | 4 |  |  |
|  |  |  |  |  |  |  | Cuts and wounds | 8 |  |  |
|  |  |  |  |  |  |  | Dry skin | 1 |  |  |
|  |  |  |  |  |  |  | Scabies | 1 |  |  |
|  | *Anthemis tinctoria* L. | *dzeltenā ilzīte* | Flowers | Decoction, tea, tincture | O. ad. | Digestive | Jaundice | 15 |  |  |
|  | *Arctium lappa* L. | *lielais diždadzis* | Leaves, roots | Decoction, juice, raw material | Ext., O. ad. | Digestive | Dysentery | 1 | F: EMA/HMPC/246763/2009 (root)  Seborrhoeic skin conditions  Loss of appetite^#^  Adjuvant in minor urinary complaints^#^ | × (root) |
|  |  |  |  |  |  |  | Toothache | 1 |  |  |
|  |  |  |  |  |  | General and unspecified | Swelling | 1 |  |  |
|  |  |  |  |  |  | Skin | Boils | 1 |  |  |
|  |  |  |  |  |  |  | Cuts and wounds | 1 |  |  |
|  |  |  |  |  |  |  | Hair loss | 1 |  |  |
|  | *Arnica montana* L. | *kalnu arnika* | Flowers | Decoction, juice, tea, tincture | Ext., O. ad. | Digestive | Hernia | 3 | F: EMA/HMPC/198793/2012  Bruises, sprains, localised muscular pain | × (flower) |
|  |  |  |  |  |  | Skin | Cuts and wounds | 1 |  |  |
|  | *Artemisia abrotanum* L. | *dievkociņš* | Leaves | Decoction, raw material, tea, with urine | Ext., O. ad. | Digestive | Generalized abdominal pain | 1 |  |  |
|  |  |  |  |  |  |  | Sharp, throbbing abdominal pain | 2 |  |  |
|  |  |  |  |  |  |  | Toothache | 2 |  |  |
|  |  |  |  |  |  | Respiratory | Chest pain | 1 |  |  |
|  |  |  |  |  |  |  | Respiratory problems | 1 |  |  |
|  |  |  |  |  |  | Skin | Lice | 1 |  |  |
|  |  |  |  |  |  |  | Lichen | 2 |  |  |
|  | *Artemisia absinthium* L. | *vērmele* | Flowers | Decoction, raw material, tea, tincture | Ext., O. ad. | Digestive | Stomach function disorders | 2 | F: EMA/HMPC/751490/2016 (herb)  Loss of appetite  Mild dyspeptic/  gastrointestinal disorders | × (aerial part) |
|  |  |  |  |  |  |  | Diarrhoea | 2 |  |  |
|  |  |  |  |  |  |  | Dysentery | 3 |  |  |
|  |  |  |  |  |  |  | Generalized abdominal pain | 22 |  |  |
|  |  |  |  |  |  |  | Heartburn | 1 |  |  |
|  |  |  |  |  |  |  | Hernia | 2 |  |  |
|  |  |  |  |  |  |  | Jaundice | 1 |  |  |
|  |  |  |  |  |  |  | Nausea | 2 |  |  |
|  |  |  |  |  |  |  | Toothache | 2 |  |  |
|  |  |  |  |  |  |  | Worms | 2 |  |  |
|  |  |  |  |  |  | Endocrine, metabolic and nutritional | Loss of appetite | 2 |  |  |
|  |  |  |  |  |  | Female genital system and breast | Menstrual problems | 1 |  |  |
|  |  |  |  |  |  | General and unspecified | Bacterial infection | 2 |  |  |
|  |  |  |  |  |  |  | Fever | 3 |  |  |
|  |  |  |  |  |  |  | Swelling | 1 |  |  |
|  |  |  |  |  |  | Musculoskeletal | Cramps | 1 |  |  |
|  |  |  |  |  |  | Neurological | Headache | 1 |  |  |
|  |  |  |  |  |  | Respiratory | Chest pain | 2 |  |  |
|  |  |  |  |  |  |  | Cough | 4 |  |  |
|  |  |  |  |  |  |  | Respiratory problems | 2 |  |  |
|  |  |  |  |  |  | Skin | Boils | 1 |  |  |
|  |  |  |  |  |  |  | Cuts and wounds | 2 |  |  |
|  |  |  |  |  |  |  | Erysipelas | 1 |  |  |
|  | *Artemisia vulgaris* L. | *parastā vībotne* | Aerial parts, roots | Decoction, tea, with milk | Ext., O. ad. | Digestive | Generalized abdominal pain | 1 |  | × (aerial part) |
|  |  |  |  |  |  | Female genital system and breast | Absent menstruation | 2 |  |  |
|  |  |  |  |  |  |  | Excessive menstrual bleeding | 1 |  |  |
|  |  |  |  |  |  | Neurological | Epilepsy | 3 |  |  |
|  |  |  |  |  |  |  | Headache | 2 |  |  |
|  |  |  |  |  |  | Respiratory | Chest pain | 1 |  |  |
|  |  |  |  |  |  |  | Respiratory problems | 1 |  |  |
|  |  |  |  |  |  | Skin | Snake bite | 1 |  |  |
|  | *Calendula officinalis* L. | *ārstniecības kliņģerīte* | Flowers | Tea | O. ad. | Digestive | Diarrhoea | 1 | F: EMA/HMPC/437450/2017  Skin inflammation and minor wounds^#^  Minor inflammations in the mouth or the throat^#^ | × (flower) |
|  |  |  |  |  |  |  | Jaundice | 1 |  |  |
|  | *Carduus* L. | *dzelksnis* | Roots | Tea | O. ad. | Respiratory | Chest pain | 2 |  |  |
|  | *Cyanus segetum* Hill | *parastā rudzupuķe* | Aerial part, flowers, straw | Bath, decoction, tea | Ext., Fumigation, O. ad. | Digestive | Diarrhoea | 1 |  |  |
|  |  |  |  |  |  |  | Sharp, throbbing abdominal pain | 1 |  |  |
|  |  |  |  |  |  | Eye | Eye pain | 1 |  |  |
|  |  |  |  |  |  | General and unspecified | Fever | 2 |  |  |
|  |  |  |  |  |  |  | Tuberculosis | 2 |  |  |
|  |  |  |  |  |  | Psychological | Sleep disturbance | 2 |  |  |
|  |  |  |  |  |  | Respiratory | Chest pain | 1 |  |  |
|  |  |  |  |  |  |  | Cough | 3 |  |  |
|  | *Cichorium intybus* L. | *parastais cigoriņš* | Aerial parts, roots | Tea | O. ad. | Digestive | Nausea | 1 | F: EMA/HMPC/121816/2010 (root)  Mild digestive disorders (abdominal fullness, flatulence, slow digestion)  Loss of appetite^#^ |  |
|  |  |  |  |  |  | Respiratory | Respiratory problems | 1 |  |  |
|  | *Cirsium arvense* (L.) Scop. | *tīruma usne* | Unsp | Tea | O. ad. | Circulatory | Cardiovascular problems | 1 |  |  |
|  | *Cirsium vulgare* (Savi) Ten. | *asais dadzis* | Inflores-cence, roots, seeds | Ashes, raw material, tea | Ext., O. ad. | Digestive | Sharp, throbbing abdominal pain | 2 |  |  |
|  |  |  |  |  |  |  | Toothache | 1 |  |  |
|  |  |  |  |  |  | Respiratory | Cough | 1 |  |  |
|  |  |  |  |  |  | Skin | Herpes simplex | 1 |  |  |
|  | *Dahlia* Cav. | *dālija* | Roots | Tea | O. ad. | Digestive | Jaundice | 1 |  |  |
|  |  |  |  |  |  | Neurological | Epilepsy | 1 |  |  |
|  |  |  |  |  |  |  | Headache | 1 |  |  |
|  | *Gnaphalium uliginosum* L. | *dumbrāja zaķpēdiņa* | Aerial parts | Powder, tea | Ext., O. ad. | Respiratory | Cough | 1 |  |  |
|  |  |  |  |  |  | Skin | Lichen | 2 |  |  |
|  | *Helianthus annuus* L. | *vasaras saulgrieze* | Seeds | Oil | Ext. | Skin | Dry skin | 1 |  |  |
|  | *Helianthus tuberosus* L. | *topinambūrs* | Unsp | Unsp | Unsp | Unsp | Unsp | 1 |  |  |
|  | *Helichrysum arenarium* (L.) Moench | *dzeltenā salmene* | Flowers | Decoction, tea | O. ad. | Digestive | Colic | 1 | F: EMA/HMPC/41108/2015  Digestive disorders with a feeling of fullness and bloating | × (flower) |
|  |  |  |  |  |  |  | Jaundice | 9 |  |  |
|  |  |  |  |  |  | Musculoskeletal | Cramps | 1 |  |  |
|  |  |  |  |  |  |  | Musculoskeletal problems | 1 |  |  |
|  | *Inula helenium* L. | *helēniju ālante* | Flowers, roots | Decoction, tea, tincture | Ext., O. ad. | Digestive | Generalized abdominal pain | 4 |  | × (rhizome) |
|  |  |  |  |  |  |  | Hernia | 10 |  |  |
|  |  |  |  |  |  |  | Sharp, throbbing abdominal pain | 2 |  |  |
|  |  |  |  |  |  | General and unspecified | Frostbite | 2 |  |  |
|  |  |  |  |  |  | Neurological | Headache | 1 |  |  |
|  |  |  |  |  |  | Respiratory | Chest pain | 2 |  |  |
|  |  |  |  |  |  |  | Respiratory problems | 2 |  |  |
|  |  |  |  |  |  | Skin | Bruise/ contusion | 1 |  |  |
|  |  |  |  |  |  |  | Skin problems | 1 |  |  |
|  | *Leucanthemum vulgare* (Vaill.) Lam. | *parastā pīpene* | Flowers, leaves | Decoction, raw material, tea | Ext., O. ad. | General and unspecified | Fever | 1 |  |  |
|  |  |  |  |  |  | Musculoskeletal | Cramps | 1 |  |  |
|  |  |  |  |  |  | Skin | Cuts and wounds | 4 |  |  |
|  |  |  |  |  |  |  | Freckles | 1 |  |  |
|  | *Matricaria chamomilla* L. | *ārstniecības kumelīte* | Flowers, leaves | Bath, compresses, decoction, raw material, tea | Ext., Inhalation, O. ad. | Digestive | Constipation | 2 | F: EMA/HMPC/55843/2011 (flower)  Minor inflammation of the skin (sunburn), superficial wounds and small boils (furuncles)  Minor gastro-intestinal complaints such as bloating and minor spasms  Cough and cold  Minor ulcers and inflammations of the  mouth and throat  Irritations of skin and mucosae  in the anal and genital region | × (flower) |
|  |  |  |  |  |  |  | Diarrhoea | 1 |  |  |
|  |  |  |  |  |  |  | Generalized abdominal pain | 4 |  |  |
|  |  |  |  |  |  |  | Toothache | 2 |  |  |
|  |  |  |  |  |  | Ear | Earache | 7 |  |  |
|  |  |  |  |  |  | Endocrine, metabolic and nutritional | Feeding problem of infant/child | 1 |  |  |
|  |  |  |  |  |  | Eye | Eye pain | 8 |  |  |
|  |  |  |  |  |  | Female genital system and breast | Induced abortion | 1 |  |  |
|  |  |  |  |  |  |  | Female genital candidiasis | 1 |  |  |
|  |  |  |  |  |  | General and unspecified | Fever | 3 |  |  |
|  |  |  |  |  |  |  | Swelling | 1 |  |  |
|  |  |  |  |  |  | Musculoskeletal | Rheumatism | 1 |  |  |
|  |  |  |  |  |  | Neurological | Headache | 6 |  |  |
|  |  |  |  |  |  | Pregnancy, childbirth, family planning | Complicated labour/delivery livebirth | 2 |  |  |
|  |  |  |  |  |  |  | Post-partum problems | 3 |  |  |
|  |  |  |  |  |  | Psychological | Sleep disturbance | 1 |  |  |
|  |  |  |  |  |  |  | Feeling anxious/ nervous/ tense | 1 |  |  |
|  |  |  |  |  |  | Respiratory | Chest pain | 1 |  |  |
|  |  |  |  |  |  |  | Cough | 20 |  |  |
|  |  |  |  |  |  |  | Voice loss | 2 |  |  |
|  |  |  |  |  |  |  | Acute upper respiratory infection | 5 |  |  |
|  |  |  |  |  |  |  | Sore throat | 5 |  |  |
|  |  |  |  |  |  |  | Pneumonia | 1 |  |  |
|  |  |  |  |  |  |  | Runny nose | 3 |  |  |
|  |  |  |  |  |  | Skin | Boils | 1 |  |  |
|  |  |  |  |  |  |  | Cuts and wounds | 3 |  |  |
|  |  |  |  |  |  |  | Vernix | 1 |  |  |
|  |  |  |  |  |  | Urology | Cystitis | 1 |  |  |
|  |  |  |  |  |  |  | Kidney problems | 1 |  |  |
|  | *Scorzonera humilis* L. | *zemā raudupe* | Roots | Tincture | O. ad. | General and unspecified | Fever | 1 |  |  |
|  | *Tagetes* L. | *samtene* | Unsp | Tea | O. ad. | Neurological | Headache | 1 |  |  |
|  | *Tanacetum vulgare* L. | *parastais biškrēsliņš* | Flowers, fruits, roots | Decoction (with milk), raw material, tea (with milk), tincture | Ext., O. ad. | Digestive | Generalized abdominal pain | 2 |  |  |
|  |  |  |  |  |  |  | Hernia | 1 |  |  |
|  |  |  |  |  |  |  | Worms | 16 |  |  |
|  |  |  |  |  |  | General and unspecified | Fever | 2 |  |  |
|  |  |  |  |  |  | Musculoskeletal | Pain in joint | 1 |  |  |
|  |  |  |  |  |  | Neurological | Headache | 1 |  |  |
|  |  |  |  |  |  | Respiratory | Chest pain | 2 |  |  |
|  |  |  |  |  |  |  | Cough | 2 |  |  |
|  |  |  |  |  |  | Skin | Lichen | 1 |  |  |
|  | *Taraxacum campylodes* G.E.Haglund | *ārstniecības pienene* | Flowers, flying seeds, leaves, roots | Decoction, juice, raw material, tea | Ext., O. ad. | Digestive | Generalized abdominal pain | 2 | F: EMA/HMPC/579636/2008 (leaf)  Adjuvant in minor urinary complaints^#^  F: EMA/HMPC/212895/2008 (root with herb)  Mild digestive disorders (abdominal fullness, flatulence, slow digestion)  Loss of appetite^#^  Adjuvant in minor urinary complaints^#^ | × (root with aerial part) |
|  |  |  |  |  |  |  | Hernia | 3 |  |  |
|  |  |  |  |  |  | Respiratory | Runny nose | 1 |  |  |
|  |  |  |  |  |  | Skin | Blisters | 1 |  |  |
|  |  |  |  |  |  |  | Cuts and wounds | 1 |  |  |
|  |  |  |  |  |  |  | Lichen | 2 |  |  |
|  |  |  |  |  |  |  | Warts | 2 |  |  |
|  | *Tripleurospermum inodorum* (L.) Sch.Bip. | *nesmaržīgā suņkumelīte* | Flowers | Decoction | O. ad. | Respiratory | Sore throat | 1 |  |  |
|  | *Tussilago farfara* L. | *parastā māllēpe* | Leaves | Bath, raw material, tea | Ext., O. ad., Smoking | Digestive | Generalized abdominal pain | 1 |  | × (leaf) |
|  |  |  |  |  |  |  | Hernia | 2 |  |  |
|  |  |  |  |  |  | General and unspecified | Swelling | 4 |  |  |
|  |  |  |  |  |  | Musculoskeletal | Bone pain | 1 |  |  |
|  |  |  |  |  |  | Neurological | Headache | 2 |  |  |
|  |  |  |  |  |  | Respiratory | Cough | 3 |  |  |
|  |  |  |  |  |  | Skin | Erysipelas | 1 |  |  |
| Balsaminaceae | *Impatiens noli-tangere* L. | *meža sprigane* | Unsp | Powder | Ext. | Skin | Lichen | 1 |  |  |
| Betulaceae | *Alnus glutinosa* (L.) Gaertn. | *melnalksnis* | Bark, buds, leaves | Decoction | O. ad. | Digestive | Diarrhoea | 1 |  |  |
|  |  |  |  |  |  |  | Generalized abdominal pain | 1 |  |  |
|  |  |  |  |  |  | Respiratory | Sore throat | 1 |  |  |
|  | *Alnus incana* (L.) Moench | *baltalksnis* | Unsp | Tea | O. ad. | General and unspecified | Fever | 1 |  |  |
|  | *Alnus* Mill. | *alksnis* | Bark, fruits, leaves, twigs | Bath, decoction, powder, raw material, tea | Ext., O. ad. | Digestive | Diarrhoea | 2 |  |  |
|  |  |  |  |  |  | Ear | Earache | 1 |  |  |
|  |  |  |  |  |  | Musculoskeletal | Cramps | 1 |  |  |
|  |  |  |  |  |  | Neurological | Headache | 1 |  |  |
|  |  |  |  |  |  | Respiratory | Sore throat | 1 |  |  |
|  |  |  |  |  |  | Skin | Animal bite | 1 |  |  |
|  |  |  |  |  |  |  | Athlete's foot | 9 |  |  |
|  | *Betula* L. | *bērzs* | Bark, buds, flowers, leaves, wood, twigs | Bath, decoction, raw material, steamed material, tea, tincture, with beer, wood tar | Ext., O. ad. | Circulatory | Cardiovascular problems | 2 | F: EMA/HMPC/573241/2014 (leaf)  Adjuvant in minor urinary complaints | × (leaf) |
|  |  |  |  |  |  |  | Swollen ankles/oedema | 1 |  |  |
|  |  |  |  |  |  | Digestive | Diarrhoea | 2 |  |  |
|  |  |  |  |  |  |  | Stomach cleansing | 1 |  |  |
|  |  |  |  |  |  |  | Dysentery | 2 |  |  |
|  |  |  |  |  |  |  | Generalized abdominal pain | 6 |  |  |
|  |  |  |  |  |  |  | Sharp, throbbing pain | 2 |  |  |
|  |  |  |  |  |  |  | Toothache | 1 |  |  |
|  |  |  |  |  |  | Ear | Earache | 2 |  |  |
|  |  |  |  |  |  | Endocrine, metabolic and nutritional | Loss of appetite | 1 |  |  |
|  |  |  |  |  |  | General and unspecified | Bleeding | 1 |  |  |
|  |  |  |  |  |  |  | Fever | 1 |  |  |
|  |  |  |  |  |  |  | Tuberculosis | 2 |  |  |
|  |  |  |  |  |  | Musculoskeletal | Bone pain | 2 |  |  |
|  |  |  |  |  |  |  | Rheumatism | 2 |  |  |
|  |  |  |  |  |  | Neurological | Epilepsy | 2 |  |  |
|  |  |  |  |  |  | Pregnancy, childbirth, family planning | Complicated labour/delivery livebirth | 2 |  |  |
|  |  |  |  |  |  | Respiratory | Chest pain | 3 |  |  |
|  |  |  |  |  |  |  | Cough | 3 |  |  |
|  |  |  |  |  |  |  | Diphtheria | 1 |  |  |
|  |  |  |  |  |  | Skin | Athlete's foot | 1 |  |  |
|  |  |  |  |  |  |  | Burns | 1 |  |  |
|  |  |  |  |  |  |  | Cuts and wounds | 1 |  |  |
|  |  |  |  |  |  |  | Hair loss | 3 |  |  |
|  |  |  |  |  |  |  | Localized swelling/lump | 1 |  |  |
|  |  |  |  |  |  |  | Warts | 2 |  |  |
|  |  |  |  |  |  | Urology | Kidney problems | 2 |  |  |
|  | *Betula pendula* Roth | *āra bērzs* | Buds, leaves, twigs | Raw material, steamed material, tincture | Ext., O. ad. | Digestive | Diarrhoea | 1 |  |  |
|  |  |  |  |  |  |  | Generalized abdominal pain | 1 |  |  |
|  |  |  |  |  |  | General and unspecified | Swelling | 1 |  |  |
|  |  |  |  |  |  | Musculoskeletal | Bone pain | 1 |  |  |
|  |  |  |  |  |  | Skin | Abscess | 1 |  |  |
|  | *Corylus avellana* L*.* | *parastā lazda* | Buds, flowers | Tea | O. ad. | General and unspecified | Tuberculosis | 1 |  |  |
|  |  |  |  |  |  | Pregnancy, childbirth, family planning | Female infertility | 1 |  |  |
| Boraginaceae | *Pulmonaria obscura* Dumort. | *ārstniecības lakacis* | Unsp | Tea | O. ad. | Respiratory | Respiratory problems | 1 |  | × (aerial part) |
|  | *Symphytum* L. | *tauksakne* | Roots | Raw material | Ext. | Skin | Abscess | 1 | F: EMA/HMPC/572846/2009  Sprains and bruises^#^ | × (root of *S. officinale*) |
| Brassicaceae | *Armoracia rusticana* P.Gaertn., B.Mey. et Scherb. | *mārrutks* | Leaves, roots | Decoction, raw material, tea | Ext., O. ad. | Digestive | Toothache | 2 |  |  |
|  |  |  |  |  |  | Female genital system and breast | Absent menstruation | 1 |  |  |
|  |  |  |  |  |  | General and unspecified | Tuberculosis | 1 |  |  |
|  |  |  |  |  |  | Musculoskeletal | Rheumatism | 1 |  |  |
|  |  |  |  |  |  | Respiratory | Chest pain | 1 |  |  |
|  |  |  |  |  |  |  | Sore throat | 1 |  |  |
|  | *Brassica napus* L. | *kālis* | Seeds | Unsp | O. ad. | Pregnancy, childbirth, family planning | Complicated labour/delivery livebirth | 1 |  |  |
|  | *Brassica oleracea* L. | *dārza kāposts* | Leaves, seeds | Decoction, raw material | Ext., O. ad. | Neurological | Headache | 2 |  |  |
|  |  |  |  |  |  | Pregnancy, childbirth, family planning | Female infertility | 1 |  |  |
|  |  |  |  |  |  | Skin | Cuts and wounds | 1 |  |  |
|  | *Brassica rapa* L. | *rācenis* | Leaves, seeds, tuber | Decoction, dew, raw material, tincture | Ext., O. ad. | Digestive | Diarrhoea | 1 |  |  |
|  |  |  |  |  |  | Eye | Eye problems | 1 |  |  |
|  |  |  |  |  |  | General and unspecified | Chickenpox | 1 |  |  |
|  |  |  |  |  |  |  | Measles | 3 |  |  |
|  |  |  |  |  |  | Respiratory | Sore throat | 1 |  |  |
|  |  |  |  |  |  | Skin | Scabies | 1 |  |  |
|  | *Capsella bursa-pastoris* (L.) Medik. | *ganu plikstiņš* | Unsp | Unsp | Unsp | Pregnancy, childbirth, family planning | Female infertility | 1 | F: EMA/HMPC/262766/2010 (herb)  Heavy menstrual bleeding^#^ | × (aerial part) |
|  | *Lepidium sativum* L. | *dārza cietķērsa* | Unsp | Unsp | Unsp | Unsp | Unsp | 1 |  |  |
|  | *Raphanus raphanistrum* subsp*. sativus* (L.) Domin | *rutks* | Roots | Juice, raw material | Ext., O. ad. | Digestive | Hernia | 1 |  |  |
|  |  |  |  |  |  |  | Worms | 1 |  |  |
|  |  |  |  |  |  | General and unspecified | Tuberculosis | 1 |  |  |
|  |  |  |  |  |  | Musculoskeletal | Bone pain | 1 |  |  |
|  |  |  |  |  |  | Neurological | Headache | 2 |  |  |
|  |  |  |  |  |  | Respiratory | Chest pain | 1 |  |  |
|  |  |  |  |  |  |  | Cough | 4 |  |  |
|  |  |  |  |  |  |  | Voice loss | 1 |  |  |
|  | *Sinapis* L. | *sinepe* | Unsp | Tea, tincture, with fat | Ext. | Digestive | Toothache | 2 |  |  |
|  |  |  |  |  |  | Skin | Boils | 1 |  |  |
|  |  |  |  |  |  |  | Cuts and wounds | 1 |  |  |
| Campanulaceae | *Campanula trachelium* L. | *strēļu pulkstenīte* | Unsp | Unsp | Unsp | Unsp | Unsp | 1 |  |  |
| Cannabaceae | *Cannabis sativa* L. | *sējas kaņepe* | Aerial parts, leaves, stem | Juice, raw material | Ext., Inhalation, Smoking | Digestive | Toothache | 2 |  |  |
|  |  |  |  |  |  | General and unspecified | Toxic effect non-medicinal substance | 1 |  |  |
|  |  |  |  |  |  | Skin | Burns | 1 |  |  |
|  |  |  |  |  |  |  | Cuts and wounds | 1 |  |  |
|  | *Humulus lupulus* L. | *parastais apinis* | Flowers, roots, stem | Ashes, bath, decoction, oil, tea | Ext., Inhalation, O. ad. | Digestive | Toothache | 2 | F: EMA/HMPC/682384/2013 (strobiles)  Mental stress and sleep disorders | × (strobile, grains) |
|  |  |  |  |  |  | Neurological | Headache | 1 |  |  |
|  |  |  |  |  |  |  | Walking difficulties in children | 1 |  |  |
|  |  |  |  |  |  | Skin | Hair loss | 1 |  |  |
|  |  |  |  |  |  |  | Herpes simplex | 1 |  |  |
|  |  |  |  |  |  |  | Scabies | 1 |  |  |
| Caprifoliaceae | *Knautia arvensis* (L.) Coult. | *tīruma pēterene* | Unsp | Unsp | O. ad. | Musculoskeletal | Fracture | 1 |  |  |
|  | *Succisa pratensis* Moench | *pļavas vilkmēle* | Unsp | Unsp | Unsp | Digestive | Toothache | 1 |  |  |
|  | *Valeriana officinalis* L. | *ārstniecības baldriāns* | Flowers, roots | Bath, decoction, juice, raw material, tea, tincture | Ext., Fumigation, O. ad. | Circulatory | Cardiovascular problems | 3 | F: EMA/HMPC/150848/2015, Corr. (root)  Sleep disorders and temporary insomnia  Mental stress and mood disorders | × (root) |
|  |  |  |  |  |  | Digestive | Diarrhoea | 1 |  |  |
|  |  |  |  |  |  |  | Generalized abdominal pain | 3 |  |  |
|  |  |  |  |  |  |  | Dental problem | 1 |  |  |
|  |  |  |  |  |  |  | Toothache | 1 |  |  |
|  |  |  |  |  |  | Musculoskeletal | Cramps | 6 |  |  |
|  |  |  |  |  |  |  | Rheumatism | 2 |  |  |
|  |  |  |  |  |  | Neurological | Epilepsy | 2 |  |  |
|  |  |  |  |  |  |  | Headache | 3 |  |  |
|  |  |  |  |  |  | Pregnancy, childbirth, family planning | Complicated labour/delivery livebirth | 2 |  |  |
|  |  |  |  |  |  | Psychological | Feeling anxious/ nervous/ tense | 7 |  |  |
|  |  |  |  |  |  |  | Sleep disturbance | 3 |  |  |
|  |  |  |  |  |  | Respiratory | Chest pain | 1 |  |  |
|  |  |  |  |  |  | Skin | Lichen | 1 |  |  |
|  |  |  |  |  |  | Urology | Kidney problems | 1 |  |  |
| Caryophyllaceae | *Agrostemma githago* L. | *lauka kokalis* | Unsp | Tea | O. ad. | General and unspecified | Measles | 1 |  |  |
|  |  |  |  |  |  |  | Scarlet fever | 1 |  |  |
|  | *Dianthus deltoides* L. | *dzirkstelīte* | Unsp | Tea | O. ad. | Digestive | Dysentery | 1 |  |  |
|  |  |  |  |  |  |  | Sharp, throbbing abdominal pain | 1 |  |  |
|  | *Herniaria* L. | *trūkumzālīte* | Unsp | Unsp | Unsp | Digestive | Sharp, throbbing abdominal pain | 1 | D: EMA/HMPC/1887935/2018 (herb)  Urinary tract and genital disorders^#^ | × (aerial part of *H. glabra*) |
|  | *Silene* L. | *plaukšķene* | Unsp | Decoction | O. ad. | Digestive | Generalized abdominal pain | 1 |  |  |
|  | *Stellaria* L. | *virza* | Aerial parts | Raw material | Ext. | Neurological | Headache | 1 |  |  |
|  |  |  |  |  |  | Skin | Athlete's foot | 1 |  |  |
|  | *Stellaria media* (L.) Vill. | *parastā virza* | Aerial parts | Steamed material | Ext. | Respiratory | Respiratory problems | 1 |  |  |
|  | *Viscaria vulgaris* Bernh. | *lipīgā sveķene* | Flowers | Tincture | O. ad. | Digestive | Sharp, throbbing abdominal pain | 1 |  |  |
| Celastraceae | *Euonymus* L. | *segliņš* | Leaves | Tea | O. ad. | Respiratory | Chest pain | 1 |  |  |
|  | *Parnassia palustris* L. | *purva atālene* | Unsp | Decoction | O. ad. | Circulatory | Cardiovascular problems | 1 |  |  |
|  |  |  |  |  |  | Musculoskeletal | Cramps | 1 |  |  |
| Crassulaceae | *Sempervivum globiferum* L. | *atvašu saulrietenis* | Unsp | Juice | Ext. | Ear | Earache | 2 |  |  |
|  | *Sedum* L. | *laimiņš* | Aerial parts | Raw material | Ext. | Musculoskeletal | Rheumatism | 1 |  |  |
|  | *Sedum acre* L. | *kodīgais laimiņš* | Aerial parts | Bath, tea | Ext., O. ad. | General and unspecified | Fever | 1 |  |  |
|  |  |  |  |  |  | Neurological | Epilepsy | 1 |  |  |
|  | *Sedum maximum* (L.) Hoffm. | *lielais laimiņš* | Aerial parts | Raw material | O. ad. | Psychological | Sleep disturbance | 2 |  |  |
|  | *Sempervivum tectorum* L. | *jumtu saulrietenis* | Bulb | Juice | Ext. | Ear | Earache | 1 |  |  |
| Cucurbitaceae | *Cucumis sativus* L. | *gurķis* | Fruits, seeds | Juice, pickled, raw material | Ext., O. ad. | Digestive | Jaundice | 1 |  |  |
|  |  |  |  |  |  |  | Worms | 1 |  |  |
|  |  |  |  |  |  | Eye | Eye pain | 1 |  |  |
|  |  |  |  |  |  | Respiratory | Sore throat | 1 |  |  |
|  | *Cucurbita* L. | *ķirbis* | Fruits | Raw material | O. ad. | Urology | Kidney problems | 1 | F: EMA/HMPC/136024/2010 (seed)  Lower urinary tract symptoms related to benign prostatic hyperplasia or related to an overactive bladder^#^ | × (seed of *C. pepo*) |
| Cupressaceae | *Juniperus communis* L. | *Zviedrijas kadiķis* | Aerial parts, fruits, roots | Bath, decoction, juice, raw material, tea | Body steaming, Ext., O. ad., Sauna whisk | Digestive | Bad breath | 1 | F: EMA/HMPC/441929/2008 (berry)  Adjuvant in minor urinary complaints  Digestive disorders (dyspepsia, flatulence) | × (wood, berry) |
|  |  |  |  |  |  |  | Generalized abdominal pain | 1 |  |  |
|  |  |  |  |  |  |  | Hypersalivation | 1 |  |  |
|  |  |  |  |  |  |  | Sharp, throbbing abdominal pain | 1 |  |  |
|  |  |  |  |  |  | General and unspecified | Bacterial infection | 1 |  |  |
|  |  |  |  |  |  |  | Fever | 1 |  |  |
|  |  |  |  |  |  |  | Swelling | 9 |  |  |
|  |  |  |  |  |  |  | Tuberculosis | 1 |  |  |
|  |  |  |  |  |  | Musculoskeletal | Rheumatism | 8 |  |  |
|  |  |  |  |  |  | Neurological | Headache | 1 |  |  |
|  |  |  |  |  |  | Respiratory | Chest pain | 4 |  |  |
|  |  |  |  |  |  |  | Cough | 2 |  |  |
|  |  |  |  |  |  |  | Respiratory problems | 2 |  |  |
|  |  |  |  |  |  | Skin | Scabies | 4 |  |  |
|  |  |  |  |  |  | Urology | Bladder problems | 1 |  |  |
|  |  |  |  |  |  |  | Kidney problems | 2 |  |  |
| Cyperaceae | *Carex* L. | *grīslis* | Unsp | Unsp | O. ad. | Digestive | Generalized abdominal pain | 1 |  |  |
| Droseraceae | *Drosera rotundifolia* L. | *apaļlapu rasene* | Leaves | Dew | Ext. | Eye | Eye problems | 2 |  | × (aerial part) |
|  |  |  |  |  |  | Skin | Dry skin | 1 |  |  |
| Dryopteridaceae | *Dryopteris filix-mas*(L.) Schott | *melnā ozolpaparde* | Leaves, roots | Unsp | Ext. | Ear | Earache | 1 |  |  |
|  |  |  |  |  |  | Musculoskeletal | Rheumatism | 1 |  |  |
|  |  |  |  |  |  | Skin | Snake bite | 1 |  |  |
| Equisetaceae | *Equisetum* L. | *kosa* | Aerial part | Decoction, tea | O. ad. | Blood, blood forming organs, lymphatics, spleen | Blood problems | 1 | F: EMA/HMPC/278091/2015  Adjuvant in minor urinary complaints  Superficial wounds^#^ | × (aerial part of *E. arvense*) |
|  |  |  |  |  |  | Respiratory | Chest pain | 1 |  |  |
|  |  |  |  |  |  | Urology | Cystitis | 2 |  |  |
|  | *Equisetum hyemale* L. | *ziemzaļā kosa* | Aerial part | Decoction, tea | O. ad. | Digestive | Digestive problems | 1 |  |  |
|  |  |  |  |  |  | Respiratory | Acute upper respiratory infection | 1 |  |  |
|  | *Equisetum sylvaticum* L. | *meža kosa* | Aerial part | Tea | O. ad. | Urology | Kidney problems | 1 |  |  |
| Ericaceae | *Arctostaphylos uva-ursi* (L.) Spreng. | *parastā miltene* | Leaves | Tea | O. ad. | Circulatory | Swollen ankles/oedema | 1 | F: EMA/HMPC/750269/2016  Lower urinary tract infections (burning sensation during urination and/or frequent urination in women) | × (leaf) |
|  |  |  |  |  |  | Musculoskeletal | Rheumatism | 1 |  |  |
|  |  |  |  |  |  | Psychological | Feeling anxious/ nervous/ tense | 1 |  |  |
|  |  |  |  |  |  | Urology | Cystitis | 2 |  |  |
|  | *Calluna vulgaris* (L.) Hull | *sila virsis* | Aerial parts, flowers | Bath, tea | Ext., O. ad. | Digestive | Generalized abdominal pain | 1 |  |  |
|  |  |  |  |  |  | Musculoskeletal | Rheumatism | 1 |  |  |
|  |  |  |  |  |  | Respiratory | Respiratory problems | 1 |  |  |
|  | *Chimaphila umbellata*(L.) Nutt. | *čemuru palēks* | Unsp | Decoction, tincture | O. ad. | Digestive | Hernia | 2 |  |  |
|  | *Ledum palustre* L. | *purva vaivariņš* | Flowers, leaves | Bath, decoction, tea | Ext., O. ad. | Digestive | Bad breath | 1 |  |  |
|  |  |  |  |  |  | General and unspecified | Swelling | 1 |  |  |
|  |  |  |  |  |  |  | Tuberculosis | 2 |  |  |
|  |  |  |  |  |  | Musculoskeletal | Rheumatism | 3 |  |  |
|  |  |  |  |  |  | Psychological | Acute alcohol abuse | 2 |  |  |
|  |  |  |  |  |  | Respiratory | Chest pain | 3 |  |  |
|  |  |  |  |  |  |  | Cough | 11 |  |  |
|  |  |  |  |  |  |  | Shortness of breath | 1 |  |  |
|  |  |  |  |  |  | Skin | Cuts and wounds | 1 |  |  |
|  |  |  |  |  |  |  | Dandruff | 1 |  |  |
|  |  |  |  |  |  |  | Rash on head | 1 |  |  |
|  |  |  |  |  |  |  | Snake bite | 1 |  |  |
|  | *Oxycoccus* Hill | *dzērvene* | Fruits | Decoction, juice, raw material, tea | Ext., O. ad. | Circulatory | Cardiovascular problems | 1 |  |  |
|  |  |  |  |  |  | Digestive | Toothache | 1 |  |  |
|  |  |  |  |  |  | Ear | Earache | 1 |  |  |
|  |  |  |  |  |  | General and unspecified | Cencer | 1 |  |  |
|  |  |  |  |  |  |  | Fever | 2 |  |  |
|  |  |  |  |  |  | Respiratory | Pneumonia | 2 |  |  |
|  |  |  |  |  |  | Skin | Sunburn | 1 |  |  |
|  | *Vaccinium myrtillus* L. | *mellene* | Fruits, leaves | Jam, juice, raw material, tea | O. ad. | Digestive | Diarrhoea | 9 | F: EMA/HMPC/375808/2014 (fresh fruit)  Discomfort and heaviness of legs related to minor venous circulatory disturbances^#^  Cutaneous capillary fragility^#^  F: EMA/HMPC/678995/2013 (dried fruit)  Diarrhoea  Inflammations of the oral mucosa^#^ | × (leaf, dried fruit) |
|  |  |  |  |  |  |  | Dysentery | 1 |  |  |
|  |  |  |  |  |  |  | Generalized abdominal pain | 3 |  |  |
|  |  |  |  |  |  |  | Stomach function disorder | 2 |  |  |
|  |  |  |  |  |  |  | Toothache | 1 |  |  |
|  |  |  |  |  |  | Respiratory | Chest pain | 1 |  |  |
|  |  |  |  |  |  |  | Cough | 1 |  |  |
|  | *Vaccinium vitis-idaea* L. | *brūklene* | Flowers, fruits, leaves | Bath, decoction, jam, tea | Ext., O. ad. | Blood, blood forming organs, lymphatics, spleen | Promote formation of blood | 1 |  |  |
|  |  |  |  |  |  | Circulatory | Cold feet | 1 |  |  |
|  |  |  |  |  |  | Digestive | Sharp, throbbing abdominal pain | 1 |  |  |
|  |  |  |  |  |  | General and unspecified | Fever | 1 |  |  |
|  |  |  |  |  |  | Musculoskeletal | Bone pain | 3 |  |  |
|  |  |  |  |  |  |  | Rheumatism | 10 |  |  |
|  |  |  |  |  |  | Respiratory | Chest pain | 1 |  |  |
|  |  |  |  |  |  |  | Runny nose | 1 |  |  |
| Euphorbiaceae | *Euphorbia helioscopia* L. | *saules dievkrēsliņš* | Unsp | Decoction | O. ad. | Urology | Urinary retention | 2 |  |  |
| Fabaceae | *Lotus* L. | *vanagnadziņš* | Unsp | Unsp | Unsp | Digestive | Generalized abdominal pain | 1 |  |  |
|  | *Pisum* L. | *zirnis* | Seeds | Decoction, soaked | Ext., O. ad. | Digestive | Teething problem | 1 |  |  |
|  |  |  |  |  |  | Endocrine, metabolic and nutritional | Loss of appetite | 1 |  |  |
|  | *Senna* Mill. | *senna* | Leaves | Tea | O. ad. | Digestive | Constipation | 1 | F: EMEA/HMPC/51869/2006 Corrigendum  Constipation | × (leaf, pods of *S. angustifolia*) |
|  | *Trifolium arvense* L. | *tīruma āboliņš* | Flowers | Tea | O. ad. | Respiratory | Respiratory problems | 1 |  |  |
|  | *Trifolium aureum* Pollich | *dzeltenais āboliņš* | Flowers, leaves | Decoction, tea | O. ad. | Digestive | Generalized abdominal pain | 1 |  |  |
|  |  |  |  |  |  | Neurological | Epilepsy | 1 |  |  |
|  |  |  |  |  |  | Respiratory | Voice loss | 1 |  |  |
|  | *Trifolium* L. | *āboliņš* | Flowers | Bath, decoction | Ext., O. ad. | Digestive | Dental problem | 1 |  |  |
|  |  |  |  |  |  | Female genital system and breast | Absent menstruation | 1 |  |  |
|  |  |  |  |  |  | Respiratory | Cough | 1 |  |  |
|  |  |  |  |  |  | Urology | Kidney problems | 1 |  |  |
|  | *Trifolium pratense* L. | *pļavas āboliņš* | Flowers | Decoction, tea | O. ad. | General and unspecified | Tuberculosis | 1 |  |  |
|  |  |  |  |  |  | Respiratory | Sore throat | 1 |  |  |
|  | *Trifolium repens* L. | *ložņu āboliņš* | Flowers | Bath, decoction, tea | Ext., O. ad. | Digestive | Teething problem | 1 |  |  |
|  |  |  |  |  |  | Female genital system and breast | Female genital candidiasis | 1 |  |  |
|  |  |  |  |  |  | Respiratory | Sore throat | 2 |  |  |
|  | *Trifolium spadiceum* L. | *brūnais āboliņš* | Flowers | Tea | O. ad. | Respiratory | Cough | 1 |  |  |
|  | *Vicia faba* L. | *lauka pupa* | Flowers, stem | Juice, tea | Ext., O. ad. | Female genital system and breast | Absent menstruation | 1 |  |  |
|  |  |  |  |  |  | Skin | Scar | 1 |  |  |
|  | *Vicia* L. | *vīķis* | Unsp | Tea | O. ad. | Digestive | Generalized abdominal pain | 1 |  |  |
| Fagaceae | *Quercus robur* L. | *parastais ozols* | Bark, buds, fruits, leaves, seeds | Bath, compresses, decoction, powder, raw material, roasted material, steamed material, tea | Ext., O. ad., Smoking | Circulatory | Stroke | 1 | C: EMA/HMPC/3203/2009 (bark)  Diarrhoea  Inflammation of the oral mucosa or skin | × (bark) |
|  |  |  |  |  |  | Digestive | Diarrhoea | 16 |  |  |
|  |  |  |  |  |  |  | Dysentery | 6 |  |  |
|  |  |  |  |  |  |  | Generalized abdominal pain | 5 |  |  |
|  |  |  |  |  |  |  | Hernia | 3 |  |  |
|  |  |  |  |  |  |  | Toothache | 7 |  |  |
|  |  |  |  |  |  | Female genital system and breast | Absent menstruation | 1 |  |  |
|  |  |  |  |  |  |  | Excessive menstrual bleeding | 1 |  |  |
|  |  |  |  |  |  | Neurological | Headache | 2 |  |  |
|  |  |  |  |  |  | Pregnancy, childbirth, family planning | Complicated labour/delivery livebirth | 1 |  |  |
|  |  |  |  |  |  | Respiratory | Cough | 1 |  |  |
|  |  |  |  |  |  |  | Respiratory problems | 2 |  |  |
|  |  |  |  |  |  |  | Sore throat | 2 |  |  |
|  |  |  |  |  |  | Skin | Cuts and wounds | 2 |  |  |
| Gentianaceae | *Centaurium erythraea* Rafn | *čemuru augstiņš* | Unsp | Tea | O. ad. | Digestive | Generalized abdominal pain | 1 | F: EMA/HMPC/277493/2015 (herb)  Mild dyspeptic/  gastrointestinal disorders  Loss of appetite^#^ | × (aerial part) |
| Geraniaceae | *Pelargonium* L'Hér. | *pelargonija* | Leaves, roots | Raw material, tea | Ext. | Digestive | Sharp, throbbing abdominal pain | 1 | F: EMA/HMPC/444244/2015 (root)  Cough and cold^#^ |  |
|  |  |  |  |  |  |  | Toothache | 2 |  |  |
|  |  |  |  |  |  | Ear | Earache | 7 |  |  |
| Grossulariaceae | *Ribes nigrum* L. | *parastā upene* | Bark, buds, twigs | Tea | O. ad. | General and unspecified | Swelling | 1 | F: EMA/HMPC/745353/2016 (leaf)  Articular pain  Adjuvant in minor urinary complaints^#^ | × (leaf) |
|  |  |  |  |  |  | Musculoskeletal | Rheumatism | 1 |  |  |
|  |  |  |  |  |  | Respiratory | Chest pain | 1 |  |  |
|  |  |  |  |  |  |  | Cough | 1 |  |  |
|  |  |  |  |  |  |  | Respiratory problems | 1 |  |  |
|  |  |  |  |  |  |  | Acute upper respiratory infection | 2 |  |  |
|  | *Ribes rubrum* L. | *sarkanā jāņoga* | Fruits | Juice | O. ad. | General and unspecified | Tuberculosis | 1 |  |  |
| Hypericaceae | *Hypericum* L. | *asinszāle* | Aerial part | Tea, tincture | O. ad. | Digestive | Dysentery | 1 | P: EMA/HMPC/101304/2008  Depressive disorders^#^  P: EMEA/HMPC/745582/2009  Mental exhaustion^#^  Skin inflammation and minor wounds^#^  Gastrointestinal discomfort | × (aerial part) |
|  |  |  |  |  |  |  | Generalized abdominal pain | 1 |  |  |
|  |  |  |  |  |  | Respiratory | Cough | 1 |  |  |
|  | *Hypericum perforatum* L. | *divšķautņu asinszāle* | Aerial part | Unsp | Unsp | Digestive | Dysentery | 1 |  |  |
| Iridaceae | *Gladiolus imbricatus* L. | *jumstiņu gladiola* | Bulb | Raw material | Ext., O. ad. | Digestive | Generalized abdominal pain | 1 |  |  |
|  |  |  |  |  |  |  | Toothache | 1 |  |  |
|  | *Iris* L*.* | *skalbe* | Fruits | Tincture | Ext. | Unsp | Unsp | 1 |  | × (rhizome) |
| Lauraceae | *Laurus*L. | *laurs* | Bark, fruits, leaves | Decoction with beer, powder, tea | O. ad. | Female genital system and breast | Absent menstruation | 2 |  |  |
|  |  |  |  |  |  |  | Excessive menstrual bleeding | 1 |  |  |
|  |  |  |  |  |  | General and unspecified | Fever | 1 |  |  |
|  |  |  |  |  |  |  | Scarlet fever | 1 |  |  |
|  |  |  |  |  |  |  | Measles | 1 |  |  |
|  |  |  |  |  |  | Pregnancy, childbirth, family planning | Induced abortion | 1 |  |  |
| Lamiaceae | *Glechoma hederacea* L. | *efejlapu sētložņa* | Flowers | Bath, raw material, tea | Ext., O. ad. | Digestive | Toothache | 1 |  |  |
|  |  |  |  |  |  | General and unspecified | Scarlet fever | 1 |  |  |
|  |  |  |  |  |  |  | Swelling | 1 |  |  |
|  | *Lamium album* L. | *baltā panātre* | Unsp | Decoction, juice, tea | O. ad. | Female genital system and breast | Menstrual pain | 2 |  | × (aerial part, flower) |
|  |  |  |  |  |  |  | Absent menstruation | 1 |  |  |
|  |  |  |  |  |  |  | Excessive menstrual bleeding | 1 |  |  |
|  | *Leonurus cardiaca* L. | *sirds mātere* | Unsp | Tea | O. ad. | Pregnancy, childbirth, family planning | Post-partum problems | 2 | F: EMA/HMPC/127428/2010 (herb)  Nervous tension^#^  Nervous cardiac complaints (palpitations)^#^ | × (aerial part) |
|  | *Melissa officinalis* L. | *ārstniecības melisa* | Unsp | Tea | O. ad. | Digestive | Generalized abdominal pain | 1 | F: EMA/HMPC/196745/2012 (leaf)  Gastrointestinal disorders (bloating and flatulence)  Mental stress and sleep disorders^#^ | × (leaf) |
|  | *Mentha aquatica* L. | *ūdensmētra* | Aerial parts | Tea | O. ad. | Digestive | Diarrhoea | 1 |  |  |
|  | *Mentha* L. | *mētra* | Aerial parts | Tincture | O. ad. | Digestive | Generalized abdominal pain | 1 |  |  |
|  | *Mentha spicata* L. | *krūzmētra* | Aerial parts | Decoction, tea | Ext., O. ad. | General and unspecified | Fever | 1 |  | × (leaf) |
|  |  |  |  |  |  | Skin | Hair loss | 1 |  |  |
|  | *Mentha x piperita* L. | *piparmētra* | Leaves, roots | Raw material, tea | Ext., O. ad. | Digestive | Diarrhoea | 1 | F: EMEA/HMPC/193909/2007 (leaf)  Gastrointestinal disorders (dyspepsia and flatulence) | × (leaf) |
|  |  |  |  |  |  |  | Generalized abdominal pain | 1 |  |  |
|  |  |  |  |  |  |  | Toothache | 2 |  |  |
|  |  |  |  |  |  | General and unspecified | Fever | 2 |  |  |
|  |  |  |  |  |  | Male genital system | Male sexual function problems | 1 |  |  |
|  |  |  |  |  |  | Neurological | Headache | 1 |  |  |
|  |  |  |  |  |  | Respiratory | Chest pain | 1 |  |  |
|  |  |  |  |  |  |  | Acute upper respiratory infection | 2 |  |  |
|  | *Nepeta cataria* L. | *kaķumētra* | Unsp | Tea | O. ad. | Pregnancy, childbirth, family planning | Lactation problems | 1 |  |  |
|  | *Origanum* L. | *raudene* | Unsp | With beer | O. ad. | Female genital system and breast | Absent menstruation | 1 |  |  |
|  | *Prunella vulgaris* L. | *parastā brūngalvīte* | Unsp | Tea | O. ad. | Digestive | Diarrhoea | 1 |  |  |
|  |  |  |  |  |  | Respiratory | Sore throat | 1 |  |  |
|  | *Salvia pratensis* L. | *pļavas salvija* | Unsp | Unsp | Unsp | General and unspecified | Fever | 1 |  | × (leaf of *S. officinalis*) |
|  |  |  |  |  |  | Psychological | Acute alcohol abuse | 2 |  |  |
|  |  |  |  |  |  |  | Feeling anxious/ nervous/ tense | 1 |  |  |
|  | *Satureja hortensis* L. | *pupumētra* | Unsp | Tea | O. ad. | General and unspecified | Swelling | 1 |  |  |
|  | *Thymus* L. | *mārsils* | Aerial parts | Decoction, tea | Ext., O. ad. | Neurological | Headache | 1 | F: EMA/HMPC/342332/2013  Expectorant in cough associated with cold |  |
|  |  |  |  |  |  | Respiratory | Cough | 1 |  |  |
|  |  |  |  |  |  |  | Chest pain | 2 |  |  |
|  |  |  |  |  |  | Skin | Bruise/ contusion | 1 |  |  |
|  | *Thymus pulegioides L.* | *lielais mārsils* | Aerial parts | Tea | O. ad. | Digestive | Generalized abdominal pain | 1 |  |  |
|  |  |  |  |  |  |  | Stomach function disorder | 1 |  |  |
|  | *Thymus serpyllum* L. | *mazais mārsils* | Aerial parts | Unsp | Unsp | Unsp | Unsp | 1 |  | × (aerial part) |
| Linaceae | *Linum catharticum* L. | *pļavas liniņš* | Flowers | Tincture | Ext. | Skin | Abscess | 2 |  |  |
|  |  |  |  |  |  |  | Bruise/ contusion | 2 |  |  |
|  | *Linum usitatissimum* L*.* | *sējas lins* | Seeds | Decoction, tea, with pork fat | Ext., O. ad. | Digestive | Dysentery | 1 | F: EMA/HMPC/377675/2014  Constipation^#^  Gastrointestinal discomfort^#^ | × (seed) |
|  |  |  |  |  |  | General and unspecified | Measles | 1 |  |  |
|  |  |  |  |  |  | Respiratory | Chest pain | 1 |  |  |
|  |  |  |  |  |  |  | Sore throat | 2 |  |  |
|  |  |  |  |  |  | Skin | Hair loss | 1 |  |  |
| Lobariaceae | *Lobaria pulmonaria* (L.) Hoffm. | *parastais plaukšķērpis* | Thallus | Decoction, tea | O. ad. | General and unspecified | Tuberculosis | 1 |  |  |
|  |  |  |  |  |  | Respiratory | Respiratory problems | 2 |  |  |
| Lycopodiaceae | *Huperzia selago* (L.) Bernh. ex Schrank & Mart. | *apdzira* | Aerial parts, spores | Decoction, decoction with beer, tea | Ext., O. ad. | Digestive | Hernia | 2 |  |  |
|  |  |  |  |  |  |  | Induces vomiting | 7 |  |  |
|  |  |  |  |  |  | General and unspecified | Leprosy | 1 |  |  |
|  |  |  |  |  |  | Skin | Dandruff | 2 |  |  |
|  |  |  |  |  |  |  | Hair loss | 1 |  |  |
|  |  |  |  |  |  |  | Lice | 3 |  |  |
|  |  |  |  |  |  |  | Scabies | 2 |  |  |
|  | *Lycopodium* L. | *staipeknis* | Spores | Powder | Ext. | General and unspecified | Bleeding | 2 |  | × (aerial part) |
|  |  |  |  |  |  | Skin | Cuts and wounds | 3 |  |  |
|  |  |  |  |  |  |  | Diaper rash | 2 |  |  |
|  |  |  |  |  |  |  | Athlete's foot | 1 |  |  |
|  | *Lycopodium clavatum* L. | *vālīšu staipeknis* | Spores | Powder | Ext. | Skin | Burns | 1 |  |  |
| Malvaceae | *Malva*L. | *malva* | Flowers | Tea | O. ad. | Unsp | Unsp | 1 | F: EMA/HMPC/749511/2016  Oral or pharyngeal irritation and associated dry cough^#^  Gastrointestinal discomfort^#^ | × (flower, leaf of *M. sylvestris*) |
|  | *Tilia* sp. L. | *liepa* | Bark, flowers, leaves, sapwood | Bath, compresses, decoction, tea | Ext., O. ad. | General and unspecified | Fever | 6 | F: EMA/HMPC/337066/2011 (flower)  Common cold  Mental stress | × (flower of *T. cordata*) |
|  |  |  |  |  |  |  | Tuberculosis | 1 |  |  |
|  |  |  |  |  |  | Psychological | Nightmares | 1 |  |  |
|  |  |  |  |  |  | Respiratory | Acute upper respiratory infection | 4 |  |  |
|  |  |  |  |  |  |  | Chest pain | 6 |  |  |
|  |  |  |  |  |  |  | Cough | 4 |  |  |
|  |  |  |  |  |  |  | Respiratory problems | 6 |  |  |
|  |  |  |  |  |  | Skin | Burns | 2 |  |  |
| Menyanthaceae | *Menyanthes trifoliata* L. | *trejlapu puplaksis* | Aerial parts, leaves, roots | Bath, decoction, juice, tea | Ext., O. ad. | Circulatory | Swollen ankles/oedema | 2 | D: EMA/HMPC/187996/2018 (leaf)  Gastrointestinal disorders  Loss of appetite^#^ | × (leaf) |
|  |  |  |  |  |  | Digestive | Diarrhoea | 1 |  |  |
|  |  |  |  |  |  |  | Generalized abdominal pain | 4 |  |  |
|  |  |  |  |  |  |  | Hernia | 2 |  |  |
|  |  |  |  |  |  | General and unspecified | Fever | 1 |  |  |
|  |  |  |  |  |  |  | Swelling | 2 |  |  |
|  |  |  |  |  |  |  | Tuberculosis | 3 |  |  |
|  |  |  |  |  |  | Neurological | Headache | 1 |  |  |
|  |  |  |  |  |  | Respiratory | Chest pain | 1 |  |  |
|  |  |  |  |  |  |  | Cough | 4 |  |  |
|  |  |  |  |  |  |  | Respiratory problems | 4 |  |  |
|  |  |  |  |  |  | Skin | Cuts and wounds | 1 |  |  |
|  |  |  |  |  |  |  | Athlete's foot | 1 |  |  |
| Myrtaceae | *Myrtus* L*.* | *mirte* | Leaves | Decoction, tea, with milk | Ext., O. ad. | Digestive | Generalized abdominal pain | 1 |  |  |
|  |  |  |  |  |  |  | Hernia | 1 |  |  |
|  |  |  |  |  |  |  | Toothache | 1 |  |  |
|  |  |  |  |  |  | Musculoskeletal | Cramps | 2 |  |  |
|  |  |  |  |  |  | Neurological | Epilepsy | 1 |  |  |
|  |  |  |  |  |  |  | Hair loss | 1 |  |  |
| Nymphaeaceae | *Nuphar lutea* (L.) Sm | *dzeltenā lēpe* | Leaves | Bath, raw material | Ext. | Neurological | Headache | 3 |  |  |
|  |  |  |  |  |  | Skin | Vernix | 4 |  |  |
| Oleaceae | *Fraxinus excelsior* L. | *parastais osis* | Flowers, leaves, twigs | Ashes, tea | O. ad. | Neurological | Headache | 2 | F: EMA/HMPC/239271/2011 (leaf)  Adjuvant in minor urinary complaints  Articular pain^#^ |  |
|  |  |  |  |  |  | Urology | Kidney problems | 1 |  |  |
|  | *Syringa* L. | *ceriņš* | Flowers, twigs | Steamed material, tea | Ext., O. ad. | Digestive | Diarrhoea | 1 |  |  |
|  |  |  |  |  |  |  | Teething problem | 1 |  |  |
|  |  |  |  |  |  | General and unspecified | Measles | 1 |  |  |
|  |  |  |  |  |  |  | Scarlet fever | 1 |  |  |
|  |  |  |  |  |  | Neurological | Headache | 1 |  |  |
|  |  |  |  |  |  | Respiratory | Cough | 3 |  |  |
| Orchidaceae | *Dactylorhiza* Neck. Ex Nevski | *dzegužpirkstīte* | Roots | Tincture | O. ad. | Pregnancy, childbirth, family planning | Female contraception | 1 |  |  |
|  |  |  |  |  |  |  | Induced abortion | 1 |  |  |
|  |  |  |  |  |  |  | Labour pain | 1 |  |  |
|  | *Dactylorhiza incarnata* (L.) Soó | *stāvlampu dzegužpirkstīte* | Roots | Unsp | O. ad. | Pregnancy, childbirth, family planning | Female infertility | 1 |  |  |
|  | *Dactylorhiza maculata* (L.) Soó | *plankumainā dzegužpirkstīte* | Roots | Raw material, tincture | Ext., O. ad. | Digestive | Generalized abdominal pain | 3 |  |  |
|  |  |  |  |  |  |  | Toothache | 2 |  |  |
| Oxalidaceae | *Oxalis* L. | *zaķskābene* | Leaves | Juice | Ext. | Digestive | Toothache | 1 |  |  |
| Paeoniaceae | *Paeonia* L. | *peonija* | Flowers | Tea | O. ad. | Digestive | Diarrhoea | 1 |  | × (flower of *P. officinalis* |
|  |  |  |  |  |  | Musculoskeletal | Cramps | 1 |  |  |
|  |  |  |  |  |  | Respiratory | Chest pain | 1 |  |  |
| Papaveraceae | *Chelidonium majus* L. | *lielā strutene* | Aerial parts | Juice, tea | Ext., O. ad. | Digestive | Diarrhoea | 1 |  | × (aerial part) |
|  |  |  |  |  |  | Eye | Eye problems | 1 |  |  |
|  |  |  |  |  |  | Skin | Lichen | 2 |  |  |
|  |  |  |  |  |  |  | Warts | 2 |  |  |
|  | *Fumaria officinalis* L. | *ārstniecības matuzāle* | Unsp | Decoction, tea | Ext. | Skin | Dandruff | 2 | F: EMA/HMPC/574766/2010 (herb)  Increase bile flow for the relief of symptoms of indigestion (sensation of fullness, flatulence, slow digestion)^#^ | × (aerial part) |
|  |  |  |  |  |  |  | Hair loss | 1 |  |  |
|  | *Papaver* L. | *magone* | Seeds | Decoction with milk, raw material, tea, tea with milk | O. ad. | Digestive | Dysentery | 1 |  | × (flower of *P. rhoes*) |
|  |  |  |  |  |  | General and unspecified | Measles | 1 |  |  |
|  |  |  |  |  |  | Psychological | Sleep disturbance | 6 |  |  |
| Parmeliaceae | *Cetraria islandica* (L.) Ach. | *Islandes ķērpis* | Thallus | Decoction | O. ad. | General and unspecified | Tuberculosis | 1 | F: EMA/HMPC/678891/2013  Oral or pharyngeal irritation and associated dry cough  Loss of appetite^#^ | × (thallus) |
|  |  |  |  |  |  | Respiratory | Respiratory problems | 1 |  |  |
| Pinaceae | *Picea abies*(L.) H.Karst. | *parastā egle* | Bark, buds, cones, leaves, resin, seeds, shoots | Bath, compresses, decoction, raw material, tea, with milk, with vinegar | Ext., O. ad. | Digestive | Dysentery | 1 |  |  |
|  |  |  |  |  |  | General and unspecified | Bleeding | 2 |  |  |
|  |  |  |  |  |  |  | Tuberculosis | 2 |  |  |
|  |  |  |  |  |  | Musculoskeletal | Rheumatism | 4 |  |  |
|  |  |  |  |  |  | Neurological | Headache | 3 |  |  |
|  |  |  |  |  |  | Respiratory | Chest pain | 2 |  |  |
|  |  |  |  |  |  |  | Cough | 1 |  |  |
|  |  |  |  |  |  |  | Respiratory problems | 2 |  |  |
|  |  |  |  |  |  |  | Sore throat | 6 |  |  |
|  |  |  |  |  |  |  | Runny nose | 1 |  |  |
|  |  |  |  |  |  | Skin | Abscess | 3 |  |  |
|  |  |  |  |  |  |  | Cuts and wounds | 5 |  |  |
|  |  |  |  |  |  |  | Scabies | 1 |  |  |
|  |  |  |  |  |  |  | Athlete's foot | 1 |  |  |
|  | *Pinus sylvestris* L. | *parastā priede* | Bark, buds, leaves, resin, seeds, shoots, twigs, wood | Bath, compresses, decoction, fermented material, juice, powder, raw material, tea | Ext., Inhalation, O. ad. | Digestive | Diarrhoea | 1 |  |  |
|  |  |  |  |  |  |  | Generalized abdominal pain | 1 |  |  |
|  |  |  |  |  |  | Endocrine, metabolic and nutritional | Loss of appetite | 1 |  |  |
|  |  |  |  |  |  | General and unspecified | Tuberculosis | 6 |  |  |
|  |  |  |  |  |  | Musculoskeletal | Rheumatism | 6 |  |  |
|  |  |  |  |  |  | Neurological | Headache | 1 |  |  |
|  |  |  |  |  |  | Respiratory | Acute upper respiratory infection | 3 |  |  |
|  |  |  |  |  |  |  | Chest pain | 1 |  |  |
|  |  |  |  |  |  |  | Cough | 1 |  |  |
|  |  |  |  |  |  |  | Respiratory problems | 4 |  |  |
|  |  |  |  |  |  |  | Sore throat | 3 |  |  |
|  |  |  |  |  |  | Skin | Cuts and wounds | 1 |  |  |
|  |  |  |  |  |  |  | Freckles | 1 |  |  |
|  |  |  |  |  |  |  | Warts | 1 |  |  |
|  |  |  |  |  |  | Urology | Kidney problems | 1 |  |  |
| Plantaginaceae | *Plantago* L. | *ceļteka* | Inflores-cence, leaves | Decoction, juice, raw material, tea, tincture | Ext., O. ad. | Circulatory | Heart pain | 1 | F: EMA/HMPC/437858/2010 (leaf)  Oral or pharyngeal irritations and associated dry cough | × (aerial part, leaf) |
|  |  |  |  |  |  | Digestive | Generalized abdominal pain | 3 |  |  |
|  |  |  |  |  |  |  | Toothache | 2 |  |  |
|  |  |  |  |  |  | General and unspecified | Bleeding | 7 |  |  |
|  |  |  |  |  |  |  | Fever | 4 |  |  |
|  |  |  |  |  |  |  | Swelling | 6 |  |  |
|  |  |  |  |  |  | Neurological | Dizziness | 1 |  |  |
|  |  |  |  |  |  |  | Headache | 1 |  |  |
|  |  |  |  |  |  | Respiratory | Acute upper respiratory infection | 1 |  |  |
|  |  |  |  |  |  |  | Respiratory problems | 1 |  |  |
|  |  |  |  |  |  | Skin | Abscess | 3 |  |  |
|  |  |  |  |  |  |  | Athlete's foot | 5 |  |  |
|  |  |  |  |  |  |  | Cuts and wounds | 7 |  |  |
|  |  |  |  |  |  |  | Lichen | 1 |  |  |
|  | *Plantago lanceolata* L. | *šaurlapu ceļteka* | Leaves | Raw material | Ext. | General and unspecified | Swelling | 1 |  |  |
|  |  |  |  |  |  | Skin | Abscess | 1 |  |  |
|  |  |  |  |  |  |  | Cuts and wounds | 2 |  |  |
|  | *Plantago major* L. | *lielā ceļteka* | Inflores-cence, leaves | Decoction, raw material | Ext., O. ad. | Digestive | Diarrhoea | 1 |  |  |
|  |  |  |  |  |  | General and unspecified | Fever | 4 |  |  |
|  |  |  |  |  |  |  | Swelling | 2 |  |  |
|  |  |  |  |  |  | Skin | Cuts and wounds | 4 |  |  |
|  |  |  |  |  |  |  | Localized swelling/lump | 1 |  |  |
| Poaceae | *Agropyron* Gaertn. | *vārpata* | Roots | Decoction, powder, tea | O. ad. | Digestive | Sharp, throbbing abdominal pain | 1 | F: EMA/HMPC/563408/2010 (rhizome)  Adjuvant in minor urinary complaints^#^ | × (rhizome of *A. repens*) |
|  |  |  |  |  |  | Musculoskeletal | Cramps | 2 |  |  |
|  |  |  |  |  |  | Urology | Incontinence urine | 1 |  |  |
|  | *Alopecurus*L. | *lapsaste* | Unsp | Tea | O. ad. | Digestive | Dysentery | 1 |  |  |
|  | *Avena* L. | *auza* | Fruits, straw | Bath, decoction, tea | Ext., Inhalation, O. ad., Smoking | Digestive | Jaundice | 1 | F: EMEA/HMPC/368600/2007 (fruit)  Skin inflammation and minor wounds^#^  F: EMEA/HMPC/202966/2007 (herb)  Mental stress and sleep disorders^#^ | × (aerial part of *A. sativa*) |
|  |  |  |  |  |  |  | Toothache | 1 |  |  |
|  |  |  |  |  |  | Musculoskeletal | Rheumatism | 1 |  |  |
|  |  |  |  |  |  | Neurological | Walking difficulties in children | 1 |  |  |
|  |  |  |  |  |  | Respiratory | Chest pain | 1 |  |  |
|  |  |  |  |  |  |  | Cough | 9 |  |  |
|  |  |  |  |  |  |  | Acute upper respiratory infection | 2 |  |  |
|  |  |  |  |  |  |  | Voice loss | 1 |  |  |
|  | *Hordeum* L. | *miezis* | Green shoots | Decoction | O. ad. | Digestive | Jaundice | 1 |  |  |
|  | *Phalaris arundinacea* L. | *parastais miežubrālis* | Leaves | Tea | O. ad. | Digestive | Generalized abdominal pain | 1 |  |  |
|  | *Secale* L. | *rudzi* | Flowers, green shoots, spike, straw | Bath, decoction, tea, tincture, with alkaline solution, with beer, with milk | Ext., O. ad. | Digestive | Generalized abdominal pain | 7 |  |  |
|  |  |  |  |  |  | Musculoskeletal | Rheumatism | 1 |  |  |
|  |  |  |  |  |  | Pregnancy, childbirth, family planning | Labour pain | 2 |  |  |
|  |  |  |  |  |  | Respiratory | Chest pain | 1 |  |  |
|  |  |  |  |  |  |  | Cough | 6 |  |  |
|  |  |  |  |  |  |  | Runny nose | 1 |  |  |
|  |  |  |  |  |  |  | Respiratory problems | 3 |  |  |
|  |  |  |  |  |  | Skin | Corn/callosity | 1 |  |  |
|  |  |  |  |  |  |  | Lichen | 1 |  |  |
|  | *Triticum* L. | *kviesis* | Bran | Bath | Ext. | Urology | Kidney problems | 1 |  |  |
| Polygalaceae | *Polygala amarella* Crantz | *rūgtā ziepenīte* | Unsp | Bath, tea | Ext., O. ad. | Psychological | Sleep disturbance | 1 |  |  |
|  |  |  |  |  |  | Respiratory | Respiratory problems | 1 |  |  |
|  |  |  |  |  |  | Skin | Freckles | 1 |  |  |
|  |  |  |  |  |  |  | Sunburn | 1 |  |  |
| Polygonaceae | *Polygonum* L. | *sūrene* | Unsp | Raw material, tea | Ext., O. ad. | Skin | Fleas | 2 |  |  |
|  | *Polygonum*  *aviculare* L. | *maura sūrene* | Unsp | Bath, tea | Ext. | Musculoskeletal | Musculoskeletal problems | 1 |  | × (aerial part) |
|  |  |  |  |  |  | Urology | Kidney problems | 2 |  |  |
|  | *Rheum rhaponticum* L. | *dārza rabarbers* | Leaf stalks | Jam, powder, with water | O. ad. | Respiratory | Cough | 1 |  | × (root of *R. palmatum*) |
|  |  |  |  |  |  | Digestive | Diarrhoea | 1 |  |  |
|  |  |  |  |  |  |  | Generalized abdominal pain | 2 |  |  |
|  | *Rumex acetosella* L. | *mazā skābene* | Leaves | Juice | Ext. | General and unspecified | Bleeding | 1 |  |  |
|  | *Rumex crispus* L. | *cirtainā skābene* | Flowers, leaves, roots | Decoction, raw material, tea, with cream | Ext., O. ad. | Digestive | Diarrhoea | 11 |  |  |
|  |  |  |  |  |  |  | Generalized abdominal pain | 4 |  |  |
|  |  |  |  |  |  | General and unspecified | Swelling | 1 |  |  |
|  |  |  |  |  |  | Skin | Erysipelas | 1 |  |  |
|  |  |  |  |  |  |  | Scabies | 10 |  |  |
|  | *Rumex* L. | *skābene* | Unsp | Unsp | Unsp | Unsp | Unsp | 1 |  |  |
| Polytrichaceae | *Polytrichum commune* Hedw. | *parastais dzegužlins* | Tea | Unsp | Unsp | Unsp | Unsp | 1 |  |  |
| Primulaceae | *Primula farinosa* L. | *bezdelīgactiņa* | Aerial parts | Tea | O. ad. | Digestive | Generalized abdominal pain | 1 |  |  |
|  |  |  |  |  |  | Respiratory | Respiratory problems | 1 |  |  |
|  | *Primula veris L.* | *gaiļbiksīte* | Flowers | Tea | O. ad. | Respiratory | Acute upper respiratory infection | 1 | F: EMA/HMPC/136582/2012  Expectorant in cough associated with cold | × (flower, root) |
|  |  |  |  |  |  |  | Chest pain | 1 |  |  |
|  |  |  |  |  |  |  | Respiratory problems | 2 |  |  |
| Ranunculaceae | *Aconitum napellus* L. | *zilā kurpīte* | Roots | Raw material, tea | Ext. | Digestive | Sharp, throbbing abdominal pain | 1 |  |  |
|  |  |  |  |  |  |  | Toothache | 3 |  |  |
|  |  |  |  |  |  | Skin | Lichen | 1 |  |  |
|  | *Actaea spicata* L. | *vārpainā krauklene* | Aerial parts | Unsp | Ext. | Psychological | Feeling anxious/ nervous/ tense | 1 |  |  |
|  | *Anemone* L. | *vizbulis* | Roots | Tea | O. ad. | Respiratory | Chest pain | 1 |  |  |
|  | *Anemone ranunculoides* L. | *dzeltenais vizbulis* | Unsp | Tea | O. ad. | General and unspecified | Tuberculosis | 1 |  |  |
|  | *Consolida regalis* Gray | *tīruma zilausis* | Unsp | Tea | O. ad. | Digestive | Sharp, throbbing abdominal pain | 2 |  | × (flower) |
|  | *Ranunculus acris* L. | *kodīgā gundega* | Flowers, leaves | Juice, raw material | Ext. | Digestive | Toothache | 2 |  |  |
|  |  |  |  |  |  | Skin | Freckles | 1 |  |  |
|  |  |  |  |  |  |  | Scabies | 1 |  |  |
|  |  |  |  |  |  |  | Sunburn | 1 |  |  |
|  |  |  |  |  |  |  | Ulcer | 1 |  |  |
|  |  |  |  |  |  |  | Warts | 2 |  |  |
|  | *Thalictrum* L. | *saulkrēsliņš* | Unsp | Juice, tea | Ext., O. ad. | Digestive | Generalized abdominal pain | 1 |  |  |
|  |  |  |  |  |  |  | Worms | 1 |  |  |
|  |  |  |  |  |  | Skin | Dandruff | 1 |  |  |
|  |  |  |  |  |  |  | Rash on head | 1 |  |  |
|  | *Trollius europaeus* L. | *Eiropas saulpurene* | Flowers | Decoction | O. ad. | Respiratory | Cough | 1 |  |  |
| Resedaceae | *Reseda*L. | *rezēda* | Unsp | Unsp | Unsp | Skin | Rash | 1 |  |  |
| Rhamnaceae | *Frangula alnus* Mill. | *trauslais krūklis* | Bark, fruits | Decoction, raw material, tea | Ext., O. ad. | Digestive | Constipation | 2 | F: EMEA/HMPC/76307/2006 Corrigendum (bark)  Constipation | × (bark) |
|  |  |  |  |  |  |  | Flatulence | 1 |  |  |
|  |  |  |  |  |  |  | Generalized abdominal pain | 1 |  |  |
|  |  |  |  |  |  | Musculoskeletal | Rheumatism | 1 |  |  |
|  |  |  |  |  |  | Respiratory | Cough | 3 |  |  |
|  |  |  |  |  |  |  | Runny nose | 1 |  |  |
|  |  |  |  |  |  | Skin | Scabies | 3 |  |  |
|  | *Rhamnus* L. | *pabērzs* | Bark | Powder | O. ad. | Respiratory | Cough | 3 |  | × (fruit of *R. chatarticus*) |
| Rosaceae | *Alchemilla* L. | *rasaskrēsliņš* | Aerial parts, roots | Tea | O. ad. | Respiratory | Cough | 4 |  | × (aerial part of *A. vulgaris*) |
|  | *Prunus avium* (L.) L. | *saldais ķirsis* | Bark, flowers, twigs | Decoction, tea | O. ad. | Female genital system and breast | Excessive menstrual bleeding | 2 |  |  |
|  |  |  |  |  |  | Neurological | Headache | 1 |  |  |
|  |  |  |  |  |  | Respiratory | Cough | 1 |  |  |
|  | *Comarum palustre* L. | *purva vārnkāja* | Unsp | Raw material, tea | Ext., O. ad. | General and unspecified | Sweating problem | 1 |  |  |
|  |  |  |  |  |  | Musculoskeletal | Bone pain | 2 |  |  |
|  |  |  |  |  |  |  | Sprain/strain of joint | 1 |  |  |
|  |  |  |  |  |  | Neurological | Headache | 1 |  |  |
|  |  |  |  |  |  | Respiratory | Cough | 1 |  |  |
|  | *Crataegus* L. | *vilkābele* | Unsp | Tea | Unsp | Skin | Animal bite | 1 | F: EMA/HMPC/159075/2014 (leaf and flower)  Nervous cardiac complaints (palpitations, perceived exstra heart beat due to mild anxiety)^#^  Mental stress and sleep disorders^#^ | × (leaf with flower, fruit) |
|  | *Crataegus laevigata* (Poir.) DC. | *divirbuļu vilkābele* | Bark | Tea | O. ad. | Respiratory | Cough | 1 |  |  |
|  | *Filipendula*  *ulmaria* (L.) Maxim. | *parastā vīgrieze* | Flowers, roots | Bath, decoction, juice, powder, raw material, tea | Ext., O. ad. | Digestive | Diarrhoea | 2 | F: EMA/HMPC/434894/2010 (flower)  Common cold  Articular pain | × (flower) |
|  |  |  |  |  |  |  | Generalized abdominal pain | 3 |  |  |
|  |  |  |  |  |  |  | Toothache | 2 |  |  |
|  |  |  |  |  |  | Eye | Eye problems | 1 |  |  |
|  |  |  |  |  |  | Musculoskeletal | Bone pain | 1 |  |  |
|  |  |  |  |  |  | Psychological | Feeling anxious/ nervous/ tense | 1 |  |  |
|  |  |  |  |  |  | Respiratory | Cough | 1 |  |  |
|  |  |  |  |  |  | Skin | Erysipelas | 1 |  |  |
|  |  |  |  |  |  |  | Rabies | 4 |  |  |
|  |  |  |  |  |  |  | Snake bite | 2 |  |  |
|  | *Fragaria vesca* L. | *meža zemene* | Flowers, fruits | Decoction, raw material, tea | Ext., O. ad. | Neurological | Headache | 1 | F: EMA/HMPC/432278/2015 (leaf)  Adjuvant in minor urinary complaints^#^  Diarrhoea^#^ | × (leaf) |
|  |  |  |  |  |  | Respiratory | Chest pain | 1 |  |  |
|  |  |  |  |  |  |  | Cough | 2 |  |  |
|  |  |  |  |  |  | Skin | Warts | 1 |  |  |
|  | *Malus* Mill. | *ābele* | Flowers, fruit bark, fruits, leaves, wood | Wood tar, juice, raw material, tea | Ext., O. ad. | Digestive | Generalized abdominal pain | 1 |  |  |
|  |  |  |  |  |  | Eye | Eye pain | 1 |  |  |
|  |  |  |  |  |  | Neurological | Headache | 1 |  |  |
|  |  |  |  |  |  | Musculoskeletal | Rheumatism | 1 |  |  |
|  |  |  |  |  |  | Respiratory | Respiratory problems | 4 |  |  |
|  |  |  |  |  |  | Skin | Athlete's foot | 8 |  |  |
|  |  |  |  |  |  |  | Warts | 8 |  |  |
|  | *Padus avium* Mill. | *parastā ieva* | Bark, flowers, fruits, leaves, twigs | Decoction, raw material, steamed material, tea, tincture | Ext., O. ad. | Circulatory | Localized swelling/lump | 1 |  |  |
|  |  |  |  |  |  | Digestive | Constipation | 1 |  |  |
|  |  |  |  |  |  |  | Diarrhoea | 4 |  |  |
|  |  |  |  |  |  |  | Generalized abdominal pain | 1 |  |  |
|  |  |  |  |  |  |  | Toothache | 9 |  |  |
|  |  |  |  |  |  | Ear | Ear problems | 2 |  |  |
|  |  |  |  |  |  | General and unspecified | Fever | 1 |  |  |
|  |  |  |  |  |  |  | Swelling | 5 |  |  |
|  |  |  |  |  |  | Musculoskeletal | Bone pain | 1 |  |  |
|  |  |  |  |  |  | Neurological | Headache | 4 |  |  |
|  |  |  |  |  |  | Respiratory | Cough | 1 |  |  |
|  |  |  |  |  |  |  | Sore throat | 1 |  |  |
|  |  |  |  |  |  | Skin | Bruise/ contusion | 2 |  |  |
|  |  |  |  |  |  |  | Dry skin | 1 |  |  |
|  |  |  |  |  |  |  | Erysipelas | 10 |  |  |
|  |  |  |  |  |  |  | Freckles | 1 |  |  |
|  | *Potentilla* L. | *retējs* | Roots | Tea, tincture | Ext., O. ad. | Digestive | Dysentery | 1 | C: EMA/HMPC/5513/2010 (rhizome)  Diarrhoea  Inflammations of the oral mucosa |  |
|  |  |  |  |  |  |  | Generalized abdominal pain | 5 |  |  |
|  |  |  |  |  |  |  | Hernia | 1 |  |  |
|  |  |  |  |  |  |  | Sharp, throbbing abdominal pain | 1 |  |  |
|  |  |  |  |  |  |  | Toothache | 1 |  |  |
|  |  |  |  |  |  | Endocrine, metabolic and nutritional | Overeating | 1 |  |  |
|  |  |  |  |  |  | Respiratory | Respiratory problems | 2 |  |  |
|  | *Potentilla anserina* L. | *maura retējs* | Unsp | Tea | O. ad. | Digestive | Sharp, throbbing abdominal pain | 1 |  | × (aerial part) |
|  | *Potentilla erecta* (L.) Raeusch. | *stāvais retējs* | Unsp | Unsp | Unsp | Unsp | Unsp | 1 | C: EMA/HMPC/5513/2010 (rhizome)  Diarrhoea^#^  Inflammations of the oral mucosa^#^ | × (root) |
|  | *Prunus* L. | *plūme* | Flowers, resin | Raw material | O. ad. | Neurological | Headache | 1 |  | × (flower of *P. spinosa*) |
|  |  |  |  |  |  | Respiratory | Acute upper respiratory infection | 1 |  |  |
|  | *Pyrus* L. | *bumbiere* | Flowers | Unsp | Unsp | Neurological | Headache | 1 |  |  |
|  | *Rosa* L. | *roze* | Leaves | Decoction, raw material, tea | Ext., O. ad. | Circulatory | Cardiovascular problems | 1 | F: EMA/HMPC/137299/2013 (flower)  Skin inflammation  Inflammations of the oral and pharyngeal mucosa^#^ | × (pseudo-fruit, “seeds”) |
|  |  |  |  |  |  | Eye | Eye pain | 1 |  |  |
|  |  |  |  |  |  | General and unspecified | Fever | 1 |  |  |
|  |  |  |  |  |  | Skin | Erysipelas | 1 |  |  |
|  | *Rubus caesius* L. | *zilganā kazene* | Bark, roots | Decoction, tea, with cream | Ext., O. ad. | Digestive | Diarrhoea | 1 |  | × (leaf) |
|  |  |  |  |  |  | Female genital system and breast | Menstrual problems | 2 |  |  |
|  |  |  |  |  |  | Respiratory | Cough | 2 |  |  |
|  |  |  |  |  |  | Skin | Scabies | 1 |  |  |
|  | *Rubus chamaemorus* L. | *lācene* | Unsp | Tea | O. ad. | Respiratory | Cough | 2 |  |  |
|  | *Rubus idaeus* L. | *meža avene* | Flowers, fruits, leaves, stem, stem with leaves and fruits | Jam, juice, tea | Ext., O. ad., Smoking | General and unspecified | Fever | 5 | F: EMA/HMPC/44211/2012 (leaf)  Minor inflammations in the mouth or the throat  Spasms associated with menstrual periods^#^  Diarrhoea^#^ | × (leaf) |
|  |  |  |  |  |  |  | Sweating problem | 1 |  |  |
|  |  |  |  |  |  | Neurological | Headache | 1 |  |  |
|  |  |  |  |  |  | Respiratory | Chest pain | 2 |  |  |
|  |  |  |  |  |  |  | Cough | 5 |  |  |
|  |  |  |  |  |  |  | Runny nose | 1 |  |  |
|  |  |  |  |  |  |  | Acute upper respiratory infection | 7 |  |  |
|  | *Rubus saxatilis* L. | *klinšu kaulene* | Stem | Decoction | O. ad. | Musculoskeletal | Back pain | 1 |  |  |
|  | *Sorbus aucuparia* L. | *parastais pīlādzis* | Bark, buds, flowers, fruits, wood | Jam, raw material, tea | Ext., O. ad. | Digestive | Constipation | 2 |  |  |
|  |  |  |  |  |  |  | Toothache | 1 |  |  |
|  |  |  |  |  |  | Endocrine, metabolic and nutritional | Loss of appetite | 1 |  |  |
|  |  |  |  |  |  | General and unspecified | Swelling | 1 |  |  |
|  |  |  |  |  |  | Musculoskeletal | Rheumatism | 1 |  |  |
|  |  |  |  |  |  | Neurological | Headache | 1 |  |  |
|  |  |  |  |  |  | Psychological | Feeling anxious/ nervous/ tense | 1 |  |  |
|  |  |  |  |  |  | Respiratory | Cough | 2 |  |  |
| Rubiaceae | *Galium odoratum* (L.) Scop. | *smaržīgā madara* | Unsp | Tea, tincture | O. ad. | Digestive | Diarrhoea | 1 |  | × (aerial part of *G. verum*) |
|  |  |  |  |  |  |  | Generalized abdominal pain | 1 |  |  |
|  | *Carapichea ipecacuanha* (Brot.) L.Andersson | *ipekakuāna* | Roots | Unsp | O. ad. | Digestive | Diarrhoea | 1 |  | × (root) |
| Salicaceae | *Populus* L. | *apse* | Bark, buds, shoots, stem | Ashes, decoction, tea, tincture | Ext., O. ad. | Circulatory | Heart pain | 1 |  |  |
|  |  |  |  |  |  | Digestive | Generalized abdominal pain | 2 |  |  |
|  |  |  |  |  |  |  | Toothache | 1 |  |  |
|  |  |  |  |  |  | General and unspecified | Fever | 1 |  |  |
|  |  |  |  |  |  |  | Tuberculosis | 1 |  |  |
|  |  |  |  |  |  | Musculoskeletal | Back pain | 1 |  |  |
|  |  |  |  |  |  | Respiratory | Respiratory problems | 2 |  |  |
|  | *Salix caprea* L. | *blīgzna* | Bark, twigs | Raw material, tea | Ext., O. ad. | Digestive | Toothache | 1 |  |  |
|  |  |  |  |  |  | Skin | Bruise/ contusion | 1 |  |  |
|  | *Salix* L. | *kārkls* | Bark, leaves, sapwood | Bath, decoction, raw material, steamed material, tea | Ext., O. ad. | Digestive | Diarrhoea | 1 | F: EMA/HMPC/80630/2016 – Corr (bark)  Articular pain  Fever associated with common cold^#^  Headache^#^ | × (bark) |
|  |  |  |  |  |  |  | Generalized abdominal pain | 1 |  |  |
|  |  |  |  |  |  | Eye | Eye pain | 1 |  |  |
|  |  |  |  |  |  | Musculoskeletal | Bone pain | 1 |  |  |
|  |  |  |  |  |  |  | Musculoskeletal problems | 1 |  |  |
|  |  |  |  |  |  | Respiratory | Chest pain | 1 |  |  |
|  |  |  |  |  |  | Skin | Sunburn | 1 |  |  |
|  | *Salix viminalis* L. | *klūdziņu kārkls* | Unsp | Tea | O. ad. | Respiratory | Chest pain | 1 |  |  |
| Sapindaceae | *Acer* L. | *kļava* | Leaves, wood | Charcoal, raw material | Ext. | Neurological | Headache | 2 |  |  |
|  |  |  |  |  |  | Skin | Warts | 1 |  |  |
|  | *Aesculus hippocastanum* L. | *parastā zirgkastaņa* | Flowers, seeds | Baked, decoction, tea, tincture | Ext., O. ad., Smoking | Digestive | Hernia | 1 | F: EMEA/HMPC/225319/2008 (seed)  Chronic venous insufficiency^#^  Venous circulatory disturbances^#^  Bruises of oedema and haematoma^#^  F: EMA/HMPC/354156/2011 (bark)  Venous circulatory disturbances^#^  Itching and burning associated with haemorrhoids^#^ | × (bark, leaf, seed) |
|  |  |  |  |  |  |  | Toothache | 1 |  |  |
|  |  |  |  |  |  | Female genital system and breast | Female genital candidiasis | 1 |  |  |
|  |  |  |  |  |  | Musculoskeletal system disorders | Bone pain | 2 |  |  |
|  |  |  |  |  |  |  | Rheumatism | 6 |  |  |
|  |  |  |  |  |  | Respiratory | Chest pain | 1 |  |  |
|  |  |  |  |  |  |  | Cough | 1 |  |  |
|  |  |  |  |  |  |  | Runny nose | 1 |  |  |
| Saxifragaceae | *Chrysosplenium alternifolium* L. | *pamīšlapu pakrēslīte* | Unsp | With milk | O. ad. | Digestive | Generalized abdominal pain | 1 |  |  |
| Scrophulariaceae | *Euphrasia rostkoviana* Hayne | *rostkova žibulītis* | Unsp | Unsp | Unsp | Eye | Eye problems | 1 |  | × (aerial part) |
|  | *Gratiola officinalis* L. | *ārstniecības rūgtene* | Flowers | Tea | O. ad. | Digestive | Colic | 1 |  |  |
|  | *Linaria* Mill. | *vīrcele* | Unsp | Tea | O. ad. | Psychological | Nightmares | 1 |  |  |
|  | *Linaria* vulgaris Mill. | *parastā vīrcele* | Unsp | Unsp | Ext. | Skin | Lichen | 1 |  |  |
|  | *Pedicularis* L. | *jāņeglīte* | Aerial parts | Raw material | Ext. | Skin | Lice | 1 |  |  |
|  | *Verbascum thapsus* L. | *deviņvīruspēks* | Flowers, leaves | Decoction, powder, tea, tincture | Ext., O. ad. | Digestive | Generalized abdominal pain | 3 | F: EMA/HMPC/611537/2016 (flower)  Sore throat associated with dry cough and cold | × (flower of *V. densiflorum*) |
|  |  |  |  |  |  |  | Hernia | 5 |  |  |
|  |  |  |  |  |  | Female genital system and breast | Menstrual problems | 1 |  |  |
|  |  |  |  |  |  | General and unspecified | Fever | 2 |  |  |
|  |  |  |  |  |  | Male genital system | Male genital problems | 1 |  |  |
|  |  |  |  |  |  | Musculoskeletal | Rheumatism | 1 |  |  |
|  |  |  |  |  |  | Respiratory | Cough | 4 |  |  |
|  |  |  |  |  |  |  | Throat problems | 2 |  |  |
|  |  |  |  |  |  | Skin | Dandruff | 1 |  |  |
|  |  |  |  |  |  |  | Erysipelas | 1 |  |  |
|  |  |  |  |  |  |  | Hair loss | 1 |  |  |
|  |  |  |  |  |  | Urology | Incontinence urine | 2 |  |  |
|  | *Veronica beccabunga* L. | *avota veronika* | Leaves | Juice, raw material | Ext., O. ad. | Digestive | Hernia | 1 |  |  |
|  |  |  |  |  |  | General and unspecified | Swelling | 1 |  |  |
|  | *Veronica officinalis* L. | *zemteka* | Unsp | Unsp | Unsp | Musculoskeletal system disorders | Bone pain | 1 |  | × (aerial part) |
|  | *Veronica L.* | *veronika* | Unsp | Tea | O. ad. | Digestive | Generalized abdominal pain | 1 |  |  |
| Solanaceae | *Atropa belladonna* L. | *melnā velnoga* | Unsp | Unsp | Unsp | Neurological | Headache | 1 |  |  |
|  | *Datura stramonium* L. | *parastais velnābols* | Seeds | Raw material, seeds | O. ad. | Digestive | Sharp, throbbing abdominal pain | 5 |  |  |
|  |  |  |  |  |  | Neurological | Headache | 1 |  |  |
|  | *Hyoscyamus niger* L. | *driģene* | Leaves, seeds | Raw material | Ext. | Digestive | Caries | 1 |  |  |
|  |  |  |  |  |  |  | Toothache | 4 |  |  |
|  |  |  |  |  |  |  | Sharp, throbbing abdominal pain | 2 |  |  |
|  |  |  |  |  |  | Ear | Ear problems | 1 |  |  |
|  |  |  |  |  |  | General and unspecified | Fever | 1 |  |  |
|  |  |  |  |  |  | Musculoskeletal | Bone pain | 1 |  |  |
|  |  |  |  |  |  | Neurological | Headache | 1 |  |  |
|  |  |  |  |  |  | Pregnancy, childbirth, family planning | Complicated labour/delivery livebirth | 1 |  |  |
|  |  |  |  |  |  | Skin | Cuts and wounds | 1 |  |  |
|  |  |  |  |  |  |  | Rabies | 2 |  |  |
|  | *Nicotiana* L. | *tabaka* | Leaves | Ashes, decoction, powder, raw material, tincture | Ext., O. ad., Smoking | Blood, blood forming organs, lymphatics, spleen | Spleen problems | 1 |  |  |
|  |  |  |  |  |  | Digestive | Generalized abdominal pain | 2 |  |  |
|  |  |  |  |  |  |  | Toothache | 15 |  |  |
|  |  |  |  |  |  |  | Worms | 2 |  |  |
|  |  |  |  |  |  | Ear | Earache | 1 |  |  |
|  |  |  |  |  |  | Eye | Eye pain | 2 |  |  |
|  |  |  |  |  |  | General and unspecified | Swelling | 1 |  |  |
|  |  |  |  |  |  | Neurological | Headache | 1 |  |  |
|  |  |  |  |  |  | Psychological | Chronic alcohol abuse | 2 |  |  |
|  |  |  |  |  |  | Respiratory | Sore throat | 1 |  |  |
|  |  |  |  |  |  |  | Runny nose | 1 |  |  |
|  |  |  |  |  |  | Skin | Athlete's foot | 2 |  |  |
|  |  |  |  |  |  |  | Boils | 3 |  |  |
|  |  |  |  |  |  |  | Corn/callosity | 1 |  |  |
|  |  |  |  |  |  |  | Cuts and wounds | 4 |  |  |
|  |  |  |  |  |  |  | Dandruff | 1 |  |  |
|  |  |  |  |  |  |  | Erysipelas | 1 |  |  |
|  |  |  |  |  |  |  | Lice | 2 |  |  |
|  |  |  |  |  |  |  | Lichen | 6 |  |  |
|  |  |  |  |  |  |  | Scabies | 2 |  |  |
|  |  |  |  |  |  |  | Snake bite | 1 |  |  |
|  | *Solanum dulcamara* L. | *bebrukārkliņš* | Fruits, stem | Decoction, raw material, tea | Ext., O. ad., Smoking | Digestive | Hernia | 1 | F: EMA/HMPC/734361/2011 (stem)  Recurrent eczema^#^ |  |
|  |  |  |  |  |  |  | Tooth extraction | 2 |  |  |
|  |  |  |  |  |  |  | Toothache | 2 |  |  |
|  |  |  |  |  |  | Female genital system and breast | Menstrual problems | 1 |  |  |
|  |  |  |  |  |  | Psychological | Nightmares | 1 |  |  |
|  |  |  |  |  |  | Respiratory | Acute upper respiratory infection | 1 |  |  |
|  |  |  |  |  |  |  | Cough | 17 |  |  |
|  | *Solanum americanum* Mill. | *melnā naktene* | Fruits | Bath, with milk | Ext., O. ad. | Psychological | Sleep disturbance | 3 |  |  |
|  | *Solanum tuberosum* L. | *kartupelis* | Flowers, leaves, tuber | Potato water, raw material, tea | Ext., O. ad. | Endocrine, metabolic and nutritional | Loss of appetite | 3 |  |  |
|  |  |  |  |  |  | General and unspecified | Fever | 1 |  |  |
|  |  |  |  |  |  |  | Frostbite | 1 |  |  |
|  |  |  |  |  |  | Neurological | Headache | 2 |  |  |
|  |  |  |  |  |  | Skin | Athlete's foot | 1 |  |  |
|  |  |  |  |  |  |  | Burns | 1 |  |  |
|  |  |  |  |  |  |  | Lice | 1 |  |  |
|  |  |  |  |  |  |  | Lichen | 1 |  |  |
|  |  |  |  |  |  |  | Warts | 2 |  |  |
| Thymelaeaceae | *Daphne mezereum* L. | *parastā zalktene* | Fruits, twigs | Bath, raw material | Ext. | Digestive | Tooth extraction | 3 |  |  |
|  |  |  |  |  |  |  | Toothache | 8 |  |  |
|  |  |  |  |  |  | Skin | Itchy skin | 1 |  |  |
| Urticaceae | *Urtica* L. | *nātre* | Aerial parts, roots, stem | Bath, decoction, juice, raw material, tea, tincture | Ext., O. ad., Sauna whisk, Smoking | Digestive | Generalized abdominal pain | 1 | F: EMA/HMPC/508015/2007 (leaf)  Articular pain  Adjuvant in minor urinary complaints  F: EMEA/HMPC/170261/2006 (herb)  Articular pain  Seborrhoeic skin conditions  Adjuvant in minor urinary complaints  F: EMA/HMPC/461160/2008 (root)  Lower urinary tract symptoms related to benign prostatic hyperplasia | × (aerial part/leaf, fruit, root of *U. dioica*) |
|  |  |  |  |  |  |  | Sharp, throbbing abdominal pain | 1 |  |  |
|  |  |  |  |  |  |  | Hernia | 1 |  |  |
|  |  |  |  |  |  |  | Toothache | 2 |  |  |
|  |  |  |  |  |  | Female genital system and breast | Female genital candidiasis | 1 |  |  |
|  |  |  |  |  |  | General and unspecified | Swelling | 1 |  |  |
|  |  |  |  |  |  | Musculoskeletal | Rheumatism | 5 |  |  |
|  |  |  |  |  |  | Respiratory | Chest pain | 2 |  |  |
|  |  |  |  |  |  |  | Cough | 3 |  |  |
|  |  |  |  |  |  |  | Respiratory problems | 1 |  |  |
|  |  |  |  |  |  | Skin | Burns | 1 |  |  |
|  |  |  |  |  |  |  | Itchy skin | 1 |  |  |
|  | *Urtica dioica* L. | *lielā nātre* | Roots | Decoction | O. ad. | General and unspecified | Swelling | 1 |  |  |
|  | *Urtica urens* L. | *sīkā nātre* | Aerial parts, roots | Decoction, juice, raw material | Ext., O. ad., Sauna whisk | Blood, blood forming organs, lymphatics, spleen | Blood problems | 1 |  |  |
|  |  |  |  |  |  | Musculoskeletal | Bone pain | 2 |  |  |
|  |  |  |  |  |  |  | Leg pain | 1 |  |  |
|  |  |  |  |  |  |  | Rheumatism | 2 |  |  |
|  |  |  |  |  |  | Neurological | Headache | 2 |  |  |
|  |  |  |  |  |  | Respiratory | Cough | 1 |  |  |
|  |  |  |  |  |  | Skin | Abscess | 2 |  |  |
|  |  |  |  |  |  |  | Dandruff | 1 |  |  |
|  |  |  |  |  |  |  | Itchy skin | 1 |  |  |
|  |  |  |  |  |  |  | Warts | 1 |  |  |
|  |  |  |  |  |  | Urology | Urinary retention | 1 |  |  |
| Verbenaceae | *Verbena officinalis* L. | *ārstniecības verbēna* | Unsp | Raw material | Ext. | Skin | Cuts and wounds | 1 |  | × (aerial part) |
| Viburnaceae | *Sambucus nigra* L. | *melnais plūškoks* | Flowers, fruits, leaves, twigs with leaves | Decoction, tea | Ext., O. ad. | General and unspecified | Fever | 4 | F: EMA/HMPC/611512/2016 (flower)  Cough and cold | × (flower, fruit) |
|  |  |  |  |  |  |  | Tuberculosis | 1 |  |  |
|  |  |  |  |  |  | Musculoskeletal | Rheumatism | 1 |  |  |
|  |  |  |  |  |  | Respiratory | Cough | 1 |  |  |
|  |  |  |  |  |  |  | Chest pain | 1 |  |  |
|  |  |  |  |  |  |  | Acute upper respiratory infection | 1 |  |  |
|  |  |  |  |  |  | Skin | Cuts and wounds | 1 |  |  |
|  | *Viburnum opulus* L. | *parastā irbene* | Fruits | Decoction | O. ad. | General and unspecified | Swelling | 1 |  | × (bark of *V. prunifolium*) |
| Violaceae | *Viola* L. | *vijolīte* | Roots | Powder, tea, with milk | Ext., O. ad. | Circulatory | Cardiovascular problems | 1 | F: EMA/HMPC/131734/2009 (herb with flower)  Seborrhoeic skin conditions^#^ | × (flowering aerial parts of *V. tricolor*) |
|  |  |  |  |  |  | Digestive | Colic | 1 |  |  |
|  |  |  |  |  |  |  | Diarrhoea | 1 |  |  |
|  |  |  |  |  |  |  | Generalized abdominal pain | 1 |  |  |
|  |  |  |  |  |  | Female genital system and breast | Excessive menstrual bleeding | 1 |  |  |
|  |  |  |  |  |  | Musculoskeletal | Cramps | 1 |  |  |
|  |  |  |  |  |  | Neurological | Headache | 1 |  |  |
|  |  |  |  |  |  | Pregnancy, childbirth, family planning | Complicated labour/delivery livebirth | 1 |  |  |
|  |  |  |  |  |  | Respiratory | Chest pain | 1 |  |  |
|  |  |  |  |  |  |  | Cough | 3 |  |  |
|  |  |  |  |  |  |  | Respiratory complaint | 1 |  |  |
|  |  |  |  |  |  |  | Acute upper respiratory infection | 1 |  |  |
|  | *Viola arvensis* Murray | *tīruma vijolīte* | Unsp | Tea | O. ad. | Respiratory | Sore throat | 1 |  |  |
|  | *Viola tricolor* L. | *trejkrāsu vijolīte* | Unsp | Decoction, tea | O. ad. | Musculoskeletal | Cramps | 1 |  |  |
|  |  |  |  |  |  | Respiratory | Cough | 1 |  |  |
|  |  |  |  |  |  | Skin | Skin problems | 1 |  |  |
| Zingiberaceae | *Zingiber officinale* Roscoe | *ingvers* | Rhizome | Tea | O. ad. | Digestive | Generalized abdominal pain | 1 | F: EMA/HMPC/749154/2010  Motion sickness^#^  Gastrointestinal disorders (bloating and flatulence) | × (rhizome) |
